# Incremental Net Monetary Benefit of Bariatric Surgery: Systematic Review and Meta-Analysis of Cost-Effectiveness Evidences

**DOI:** 10.1007/s11695-021-05415-9

**Published:** 2021-04-24

**Authors:** Prapaporn Noparatayaporn, Montarat Thavorncharoensap, Usa Chaikledkaew, Bhavani Shankara Bagepally, Ammarin Thakkinstian

**Affiliations:** 1 Mahidol University Health Technology Assessment (MUHTA) Graduate Program, Bangkok, Thailand; 2 Pharmacy Department, Faculty of Medicine Siriraj Hospital, Mahidol University, Bangkok, Thailand; 3 Social and Administrative Pharmacy Division, Department of Pharmacy, Faculty of Pharmacy, Mahidol University, 447 Sri Ayudhaya Rd., Rajathevi, Bangkok, 10400 Thailand; 4 ICMR–National Institute of Epidemiology, Chennai, India; 5 Department of Clinical Epidemiology and Biostatistics, Faculty of Medicine Ramathibodi Hospital, Mahidol University, Bangkok, Thailand

**Keywords:** Incremental net monetary benefit, Cost-effectiveness, Bariatric surgery, Economic evaluation

## Abstract

This systematic review aimed to comprehensively synthesize cost-effectiveness evidences of bariatric surgery by pooling incremental net monetary benefits (INB). Twenty-eight full economic evaluation studies comparing bariatric surgery with usual care were identified from five databases. In high-income countries (HICs), bariatric surgery was cost-effective among mixed obesity group (i.e., obesity with/without diabetes) over a 10-year time horizon (pooled INB = $53,063.69; 95% CI $42,647.96, $63,479.43) and lifetime horizon (pooled INB = $101,897.96; 95% CI $79,390.93, $124,404.99). All studies conducted among obese with diabetes reported that bariatric surgery was cost-effective. Also, the pooled INB for obesity with diabetes group over lifetime horizon in HICs was $80,826.28 (95% CI $32,500.75, $129,151.81). Nevertheless, no evidence is available in low- and middle-income countries.

**Supplementary Information:**

The online version contains supplementary material available at 10.1007/s11695-021-05415-9.

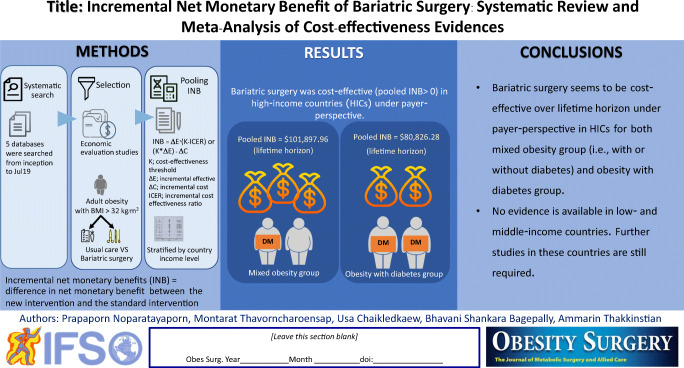

## Introduction

Bariatric surgery is an attractive treatment option for obese patients, who could not achieve weight control by conservative, non-surgical therapies. The clinical effectiveness of bariatric surgery has been well established. Evidences from previous systematic reviews consistently indicated that bariatric surgery could significantly reduce body weight and improve comorbidities, as compared with usual care (e.g., pharmacotherapy and/or lifestyle modification) [1–5]. In addition, bariatric surgery might be superior to usual care for short-term remission of diabetes mellitus [1, 6]. Nevertheless, with the growing demand for bariatric surgery, demonstrating only the clinical effectiveness is not sufficient given the limited resources for healthcare. Evidences on the value for money are strongly required to support policy decision-making. To date, there were many published systematic reviews and meta-analyses on bariatric surgery [7–9]. However, most studies were limited to the description of methodologies and economic evaluation results [9]. Existing quantitative synthesis pertained only to differences in healthcare cost pre- and post-surgery or between surgical and non-surgical approaches [7, 8]. Consequently, determining whether bariatric surgery provides value for money or in which condition it might be cost-effective is still controversial. Therefore, a meta-analysis which pools the value for money of bariatric surgery is required.

Most economic evaluation studies report incremental cost-effectiveness ratios (ICERs), which represent the ratio of incremental cost (ΔC) between the two interventions and incremental effectiveness (ΔE) between the same groups [10, 11]. The effectiveness (E) is usually measured in terms of amount of quality-adjusted life year (QALY) gained or disability-adjusted life year (DALY) averted. The ICER is then compared with the pre-defined cost-effectiveness threshold (K), the maximum amount a decision-maker is willing to pay for one QALY or DALY (e.g., £20,000 per QALY in the UK [12], or one time the GDP per capita per DALY in several countries [13]). If the ICER is less than K, the intervention is cost-effective; otherwise, it is not cost-effective. Nevertheless, the interpretation of ICER is problematic when its value is negative, which may indicate a lower cost with higher effectiveness or higher cost along with lower effectiveness of interventions. Thus, there is ambiguity in interpretation [10].

The incremental net monetary benefit (INB), which is the difference in net monetary benefit between the new intervention and the standard intervention [10], has been recently used instead of the ICER. The INB can be computed as the difference of incremental monetary benefit and incremental cost (INB = (ΔE×K) − ΔC) [10, 11]. It is relatively easier to interpret than the ICER. A positive INB (INB > 0) indicates that an intervention is cost-effective as compared with the standard intervention at the given threshold, whereas a negative INB indicates the new intervention is not cost-effective relative to the standard one [10, 11, 14, 15]. Up until now, few meta-analyses have been conducted to pool INBs [15–18]. Since there is still controversy on the value of money of bariatric surgery, we, therefore, conducted a meta-analysis which systematically reviewed and pooled INBs of bariatric surgery as compared with usual care among patients with obesity. Our main hypothesis is bariatric surgery is cost-effective for patients with obesity. Whenever possible, we examined whether bariatric surgery was cost-effective in particular type of patients (i.e,. obese with DM) and determined which specific procedures of bariatric surgery (i.e., SG, AGB, RYGB) were cost-effective when compared with usual care.

## Materials and Methods

The protocol of this systematic review was conducted and reported according to the Preferred Reporting Items for Systematic Reviews and Meta-Analyses (PRISMA) [19] (see Table S1). The review protocol was registered at the PROSPERO (registration number CRD42019142147).

### Data Sources and Search Strategies

We searched relevant studies from PubMed, Scopus, the Cochrane Central Register of Controlled Trials-Wiley library, the Cost-Effective Analysis (CEA) Registry (The CEA Registry), and the Centre for Reviews and Dissemination (CRD) since inception to July 2019 without language restrictions. We constructed search terms based on the population, intervention, outcome, and study design domains (i.e., obesity, bariatric surgery, incremental cost-effectiveness ratio, and economic evaluation) (see Table S2).

### Study Selection

Two reviewers (P.N. and M.T.) independently selected studies by screening titles and abstracts.Full texts were then reviewed based on the following criteria: any full economic evaluation study (i.e., cost-effectiveness analysis (CEA), cost–utility analysis (CUA), cost–benefit analysis (CBA)) of adult obesity (i.e., BMI > 32 kg/m^2^); compared any pair of bariatric surgery (e.g., open or laparoscopic surgeries of adjustable gastric banding (AGB), Roux-en-Y gastric bypass (RYGB), sleeve gastrectomy (SG), and a mix of these bariatric surgeries (BS)) with usual care (e.g., pharmacotherapy and/or lifestyle modification); provided sufficient data for calculating INB [17]. The studies were excluded if they studied patients with other specific diseases or their full texts were not available.

### Data Extraction

Data were extracted by two independent reviewers (P.N. and B.S.). A standardized data extraction form was developed. The data required for estimating INB were extracted: cost (C), incremental cost (ΔC), effectiveness (E), incremental effectiveness (ΔE), and ICER. These parameter values were extracted as means and dispersions (i.e., SDs, SEs, and 95% CIs). In some cases, ΔC and ΔE data were extracted from probabilistic sensitivity analyses (PSA). Authors of original studies were contacted further in case of incomplete information.

### Data Preparation

INB was calculated as follows [10, 15]: INB = (K × ΔE) − ΔC or ΔE × (K − ICER), where K is the cost-effectiveness threshold, and ΔE and ΔC are incremental effective and incremental cost, respectively. The INB variance was estimated as follows [15, 17]:

$$ \mathit{\operatorname{var}}(INB)={K}^2{\sigma}_{\varDelta E}^2+{\sigma}_{ICER}^2 $$ or $$ {K}^2{\sigma}_{\varDelta E}^2+{\sigma}_{\varDelta C}^2-2K{\rho}_{\varDelta C\varDelta E} $$, where $$ {\sigma}_{\varDelta E}^2 $$ is variance of ΔE, $$ {\sigma}_{ICER}^2 $$ is variance of ICER, $$ {\sigma}_{\varDelta C}^2 $$ is variance of ΔC, and *ρ*_*ΔCΔE*_is covariance of ΔC and ΔE. In case of incomplete data, the variance of INB was simulated according to data availability (scenarios 1 to 5), as suggested by Bagepally et al. [17] If the study did not report the cost-effectiveness threshold, the GDP per capita was used [13]. To pool data across studies, all currencies were converted to the international standard currency 2019 (international dollars; Int$) using consumer price index (CPI) and purchasing power parity (PPP) obtained from the World Economic Outlook Database (2019) [20].

### Risk-of-Bias Assessment

The quality of each study was assessed by two independent reviewers (P.N. and M.T.), using the ECOBIAS checklist [21]. Disagreement was resolved by consensus with a third reviewer (U.C.).

### Statistical Analysis

The estimated INBs were pooled across studies [15–17]. To minimize heterogeneity, studies were stratified by country income level, which was classified according to the World Bank as high-income countries (HICs) and upper-middle-income countries (UMICs) [22]. In addition, the INBs were pooled according to perspective, type of model, type of patients (i.e., mixed obesity group, which included patients with/without diabetes, obesity with diabetes group), and time horizon (i.e., lifetime, 10 years). Subgroup analysis by types of bariatric surgery (i.e., AGB, RYGB, SG, BS) was also performed. A fixed-effect model by an inverse variance method was applied for pooling if heterogeneity was not present; otherwise, a random-effect model (DerSimonian and Laird) was used [23].

The heterogeneity of INBs among the studies was assessed using the Cochrane’s *Q* statistic and *I*^2^ statistics. Publication bias was assessed using the funnel plot and Egger’s test. A contour-enhanced funnel plot was applied to distinguish publication bias from other causes of asymmetry [24].

## Results

### Study Selection

**Fig. 1:**
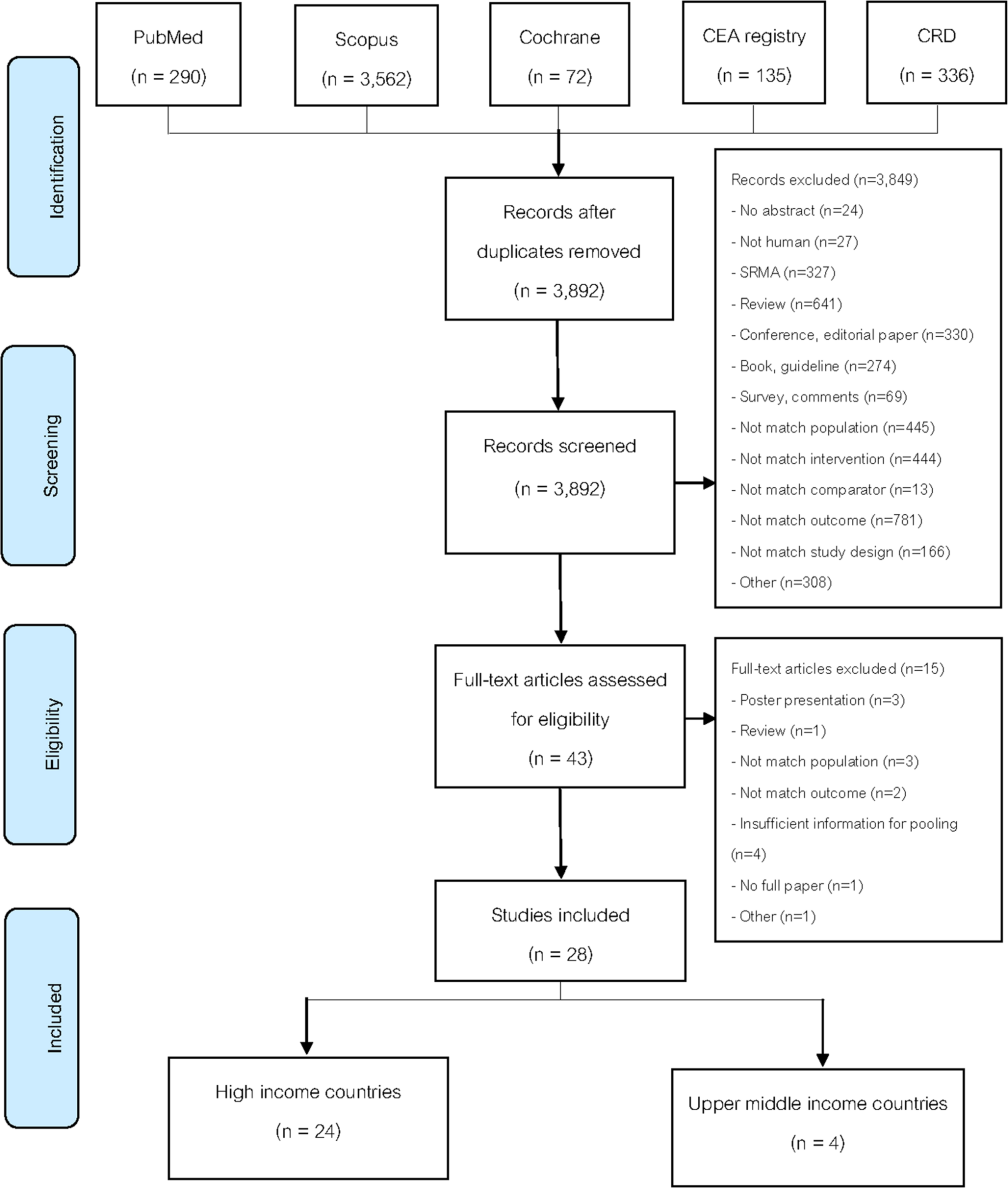
Flow diagram of the included studies

Of the 4395 identified studies, 28 were included and analyzed (see Fig. 1). The characteristics of studies are described in Tables 1 and S3. As for country income level, 24 [25–35, 37–43, 45–50] (85.7%) and 4 [36, 44, 51, 52] (14.3%) studies were conducted in HICs and UMICs, respectively. From these, 20 (71.4%) were conducted in mixed obesity group (i.e., with/without diabetes), whereas 8 [45–52] (28.6%) studies were conducted in obesity with diabetes group. All studies, except for one [52], were conducted in obese patients whose BMIs were 35 kg/m^2^ and higher. The three most common interventions were AGB (*N* = 15, 53.6%), RYGB (*N* = 20, 71.4%), and SG (*N* = 11, 39.3%).

**Table 1:** Characteristics of the included studies

Author year	Country	Characteristics of patients	Intervention	Comparator	Type of model	Perspective	Time horizon	Cycle length	Discount rate
Population: mixed obesity group (i.e., with/without diabetes)									
Craig BM 2002 [25]	USA	BMI 40–50 kg/m^2^ without chronic medical conditions	RYGB	No treatment	Decision tree	Payer	Lifetime	Not stated	3%
Campbell J 2010 [26]	USA	BMI ≥40/≥35 kg/m^2^ with comorbidity	LRYGB, LAGB	No treatment	Markov	Payer (third-party payer)	Lifetime	Not stated	3%
McEwen LN 2010 [27]	USA	BMI ≥40/≥35 kg/m^2^ with comorbidity	LRYGB 34%, ORYGB 65%, other 1%	Usual care	Not stated	Not stated	2 years, lifetime	Not stated	3%
Maklin S 2011 [28]	Finland	Morbid obesity (base case—mean BMI 47 kg/m^2^)	RYGB 68%, SG 30%, AGB 2%	Ordinary treatment	Markov decision tree	Payer (healthcare providers)	10 years	1 year	3%
Faria GR 2013 [29]	Portugal	Baseline: mean BMI 49.6 kg/m^2^	RYGB, AGB	Best medical management	Markov	Societal	Lifetime	Not stated	3%
Castilla I 2014 [30]	Spain	BMI ≥40/≥35 kg/m^2^ with comorbidity	RYGB	Usual care	Discrete-event simulation (DES)	Payer (the Spanish NHS)	5, 10 years, lifetime	Not stated	3%
Lewis L 2014 [31]	England	BMI ≥40 kg/m^2^	Opened and laparoscopic RYGB, AGB	LighterLife total	A de novo economic model	Payer (healthcare provider (the England NHS))	10 years	Not stated	3.5%
Wang BC 2014 [32]	USA	BMI ≥40/≥35 kg/m^2^ with comorbidity	LRYGB, LAGB, ORYGB	Non-surgical treatment	Decision tree and lifetime natural history model	Payer (healthcare system)	Lifetime	Not stated	3%
Borisenko O 2015 [33]	Sweden	BMI ≥40/≥35 kg/m^2^ with DM	RYGB 98%, SG 1.6%, AGB 0.4%	Optimal medical management	Markov	Payer (Swedish healthcare payer)	Lifetime	1 month	3%
Borisenko O 2017a [34]	Germany	BMI ≥40/≥35 kg/m^2^ with DM	RYGB 51%, SG 17%, AGB 33%	Conventional medical management	Markov	Payer (public payer perspective)	10 years, lifetime	1 month	3%
Borisenko O 2017b [35]	Denmark	Base case: mean BMI 42 kg/m^2^	RYGB 68.8%, SG 31%, AGB 0.2%	Optimal medical treatment	Markov	Payer (third-party payer)	10 years, lifetime	1 month	3%
Cohen RV 2017 [36]	Brazil	BMI >35 kg/m^2^ with/without DM	ORYGB >90%	Standard medical care	Markov	Payer (Brazilian Public Health System)	20 years	1 year	5%
Gulliford MC 2017 [37]	UK	Severe (BMI 35–39 kg/m^2^)/morbid obesity with/without DM	LRYGB 33%, AGB 33%, SG 33%	Standard nonsurgical management	Markov	Payer (healthcare services)	Lifetime	1 year	3.5%
James R 2017 [38]	Australia	Base case: BMI >35 kg/m^2^	AGB, RYGB, SG	Usual care	Markov	Payer (the Australian public healthcare system)	Lifetime	1 year	5%
Lucchese M 2017 [39]	Italy	BMI ≥40/≥35 kg/m^2^ with DM	RYGB 28.21%, SG 34.63%, AGB 37.15%	Optimal medical management	Markov	Payer (third-party payer)	10 years, lifetime	1 month	3%
Alsumali A 2018 [40]	USA	BMI ≥35 kg/m^2^	LRYGB, LAGB, LSG	No surgery	Markov	Payer (US health care)	Lifetime	1 year	3%
Borisenko O 2018a [41]	Belgium	BMI ≥40/≥35 kg/m^2^ with DM	RYGB 75%, SG 20%, AGB 5%	Conventional medical management (CMM)	Markov	Payer (third-party payer)	10 years, lifetime	1 month	3%
Borisenko O 2018b [42]	England	BMI ≥40/≥35 kg/m^2^ with DM	RYGB 56%, SG 22%, AGB 22%	Usual care	Markov	Payer (the National Health Service (NHS))	10 years, lifetime	1 month	3.5%
Sanchez-Santos R 2018 [43]	Spain	BMI ≥40/≥35 kg/m^2^ with DM	RYGB 76%, SG 22%, AGB 2%	Conservative management (CM)	Markov	Payer (the Spanish NHS)	10 years, lifetime	1 month	3%
Assumpção RP 2019 [44]	Brazil	BMI >40/>35 kg/m^2^ with ≥1 obesity-related comorbidities	Open gastric bypass (GBP)	Clinical treatment	Markov decision tree	Payer (the Brazilian public health system)	10 years	1 year	5%
Population: obesity with diabetes group									
Ackroyd R 2006 [45]	Germany, France, UK	BMI ≥35 kg/m^2^ with DM	LRYGB, AGB	Conventional treatment	Unspecified model	Payer	5 years	Not stated	3.5%
Anselmino M 2009 [46]	Austria, Italy, Spain	BMI ≥35 kg/m^2^ with DM	RYGB, AGB	Conventional treatment	Unspecified model	Payer	5 years	Not stated	3.5%
Ikramuddin S 2009 [47]	USA	Base case: mean BMI 48.4 kg/m^2^ with comorbidity	RYGB	Standard medical management	The CORE Diabetes Model	Payer (third-party payer)	35 years	Not stated	3%
Keating CL 2009 [48]	Australia	Base case: BMI 37 kg/m^2^ with DM (<2 years)	LAGB	Conventional therapy	Markov	Payer (healthcare system)	Lifetime	1 year	3%
Hoerger TJ 2010 [49]	USA	BMI ≥35 kg/m^2^ with DM	RYGB, AGB	Usual diabetes care	Markov	Not stated	Lifetime	1 year	3%
Pollock RF 2013 [50]	UK	Base case: BMI 37.1 kg/m^2^ with DM (average 1 year)	LAGB	Standard medical management	Version 8.0 of the core Diabetes Model (CDM)	Payer (the healthcare payer, the National Health Service)	40 years	Not stated	3.5%
Gil-Rojas Y 2019 [51]	Colombia	BMI >35 kg/m^2^ with ≥1 comorbidity	SG, RYGB	Non-surgical treatment	Markov decision tree	Payer (third-payer perspective: Colombian Health System)	5 years	1 year	5%
Viratanapanu I 2019 [52]	Thailand	BMI >32.5 kg/m^2^ with DM	BS (RYGB 61.6%)	Ordinary treatment	Markov decision tree	Payer (healthcare payer’s perspective)	50 years	1 year	3%

*BMI* body mass index, *DM* diabetes mellitus, *BS* mixed types of bariatric surgery, *AGB* adjustable gastric banding, *RYGB* Roux-en-Y gastric bypass, *SG* sleeve gastrectomy

As for methodology, all studies were CUAs. The model-based techniques were Markov model [26, 28, 29, 33–44, 47–52] (*N* = 21; 75.0%), discrete-event simulation [30] (*N* = 1; 3.6%), decision tree [25] (*N* = 1; 3.6%), decision tree and lifetime natural history model [32] (*N* = 1; 3.6%), and a de novo economic model [31] (*N* = 1; 3.6%). Two studies [45, 46] (7.1%) did not specify the type of model, while one study [27] (3.6%) did not state the technique. Most studies (*N* = 25; 89.3%) adopted a payer perspective. One study [29] adopted societal perspective and two studies [27, 49] did not clearly mention the perspective. Lifetime, 10-year, and less than 10-year horizons were respectively used in 18 [25–27, 29, 30, 32–35, 37–43, 48, 49], 10 [28, 30, 31, 34, 35, 39, 41–44], and 5 studies [27, 30, 45, 46, 51].

### INBs of Bariatric Surgery in HICs

The estimated INBs from HICs were presented separately by types of patients (see Tables S4 and S5). For mixed obesity group, bariatric surgery was found to be cost-effective in all except for three studies, which adopted 2-year [27], 5-year [30], and 10-year [31] time horizons. For obesity with diabetes group, all studies revealed that bariatric surgery was cost-effective.

### Pooled INBs of Bariatric Surgery among Mixed Obesity Group in HICs

**Fig. 2:**
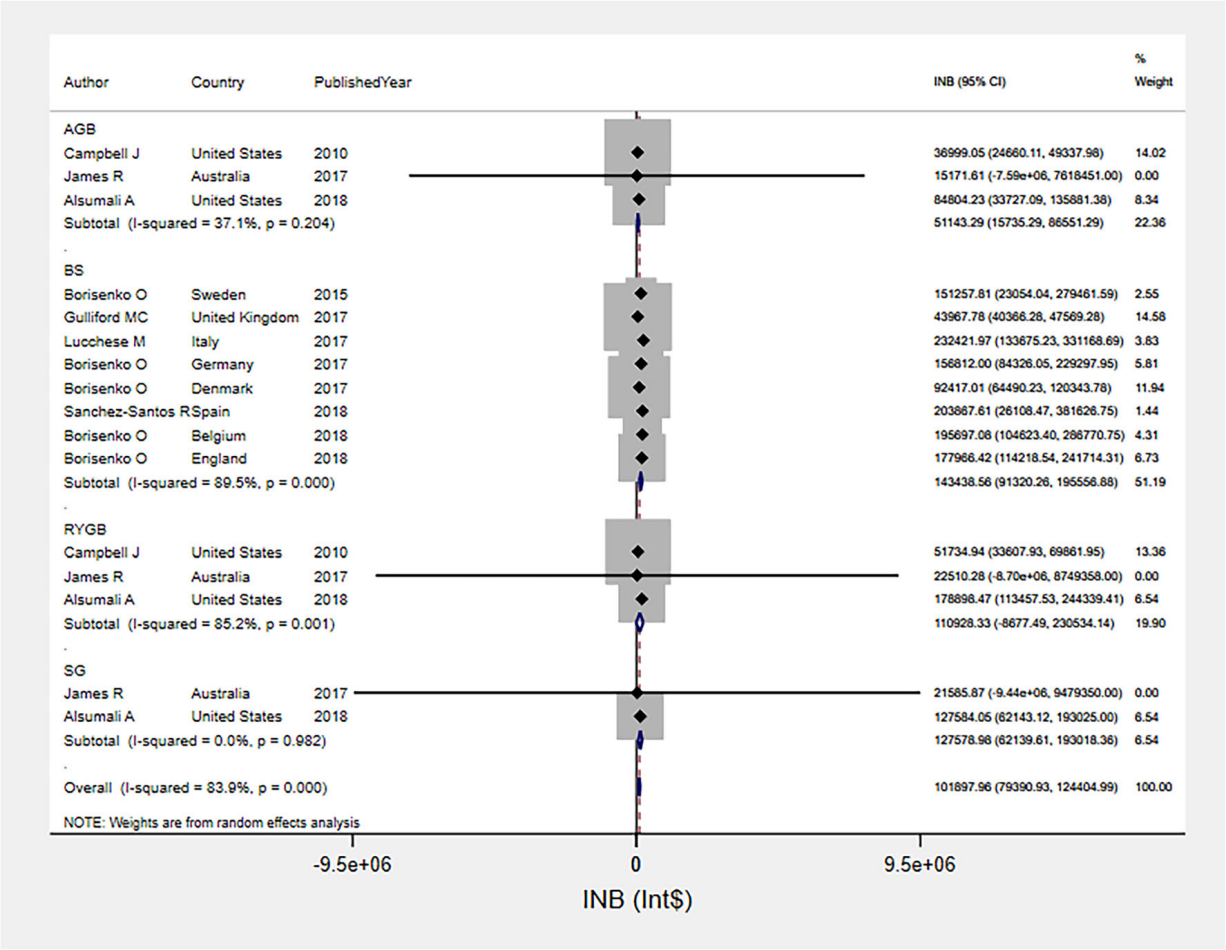
Pooled INBs of bariatric surgery among mixed obesity group in high income countries over lifetime horizon

The INBs of overall bariatric surgery versus usual care among mixed obesity group under payer perspective were highly varied across 11 studies [26, 33–35, 37–43] (*I*^2^ = 83.9%) with a pooled INB (95% CI) over lifetime horizon of $101,897.96 ($79,390.93, $124,404.99) (see Fig. 2). Subgroup analysis indicated that AGB, SG, and mixed types of BS, but not RYGB, were significantly cost-effective as compared with usual care with pooled INBs of $51,143.29 (95% CI $15,735.29, $86,551.29; *I*^2^ = 37.1%), $127,578.98 (95% CI $62,139.61, $193,018.36; *I*^2^ = 0%), $143,438.56 (95% CI $91,320.26, $195,556.88; *I*^2^ = 89.5%), and $110,928.33 (95% CI −$8,677.49, $230,534.14; *I*^2^ = 85.2.1%), respectively. A funnel plot of overall bariatric surgery effect indicated asymmetry of the funnel which contrasted to Egger’s test (coefficient = 2.11, SE = 0.47, *p* = 0.001), Fig. S1. A contour-enhanced funnel (Fig. S2) showed that asymmetry might be caused by heterogeneity.

**Fig. 3:**
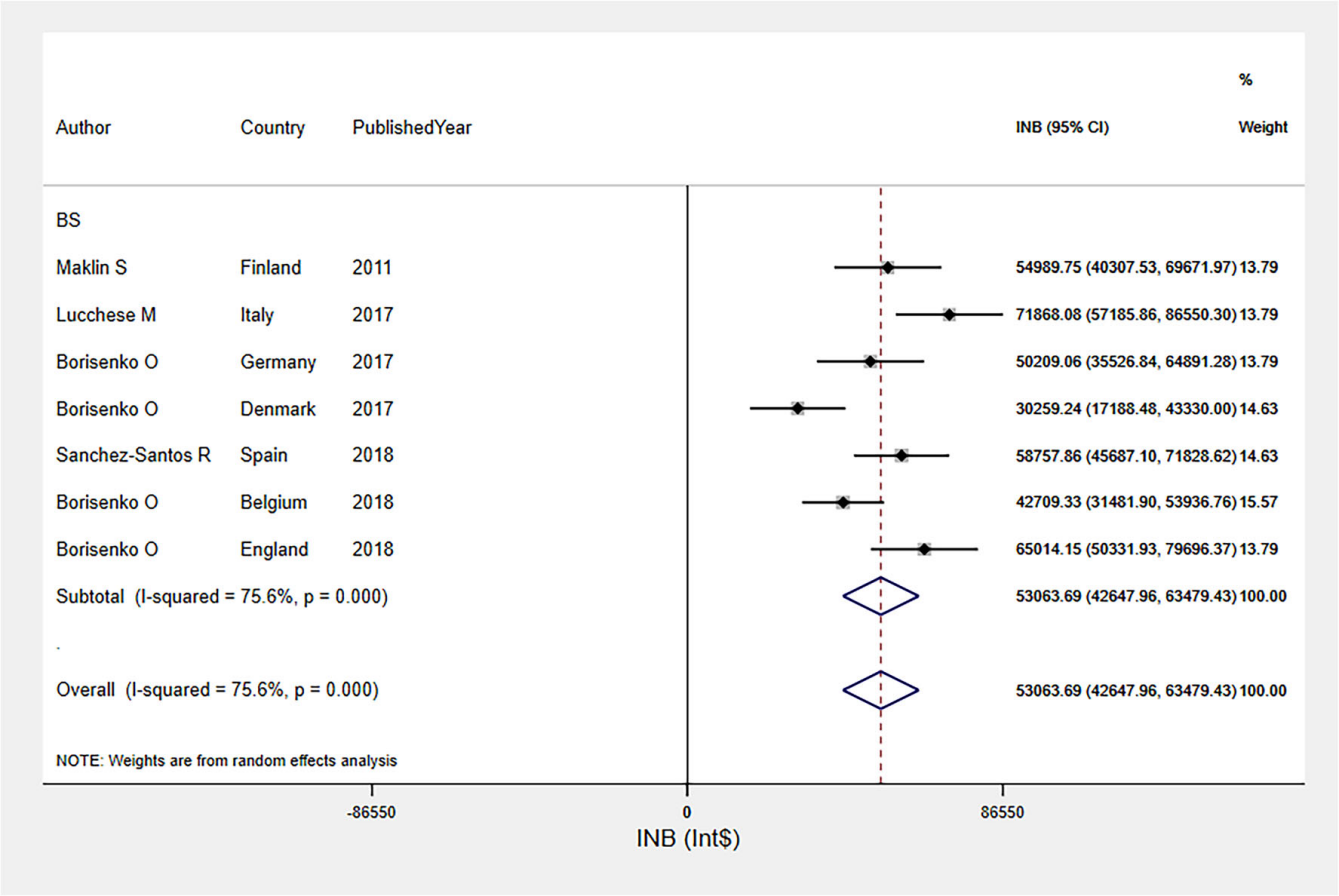
Pooled INBs of bariatric surgery among mixed obesity group in high income countries over 10-year time horizon

The INBs of bariatric surgery among mixed obesity group over a 10-year time horizon were highly varied across seven studies [28, 34, 35, 39, 41–43] (*I*^2^ = 75.6%), with a pooled INB (95% CI) of $53,063.69 ($42,647.96, $63,479.43) (see Fig. 3). A funnel plot (Fig. S3) and Egger’s test (coefficient = 11.13, SE = 6.81, *p* = 0.163) indicated no evidence of publication bias.

### Pooled INBs of Bariatric Surgery among Obesity with Diabetes in HICs

**Fig. 4:**
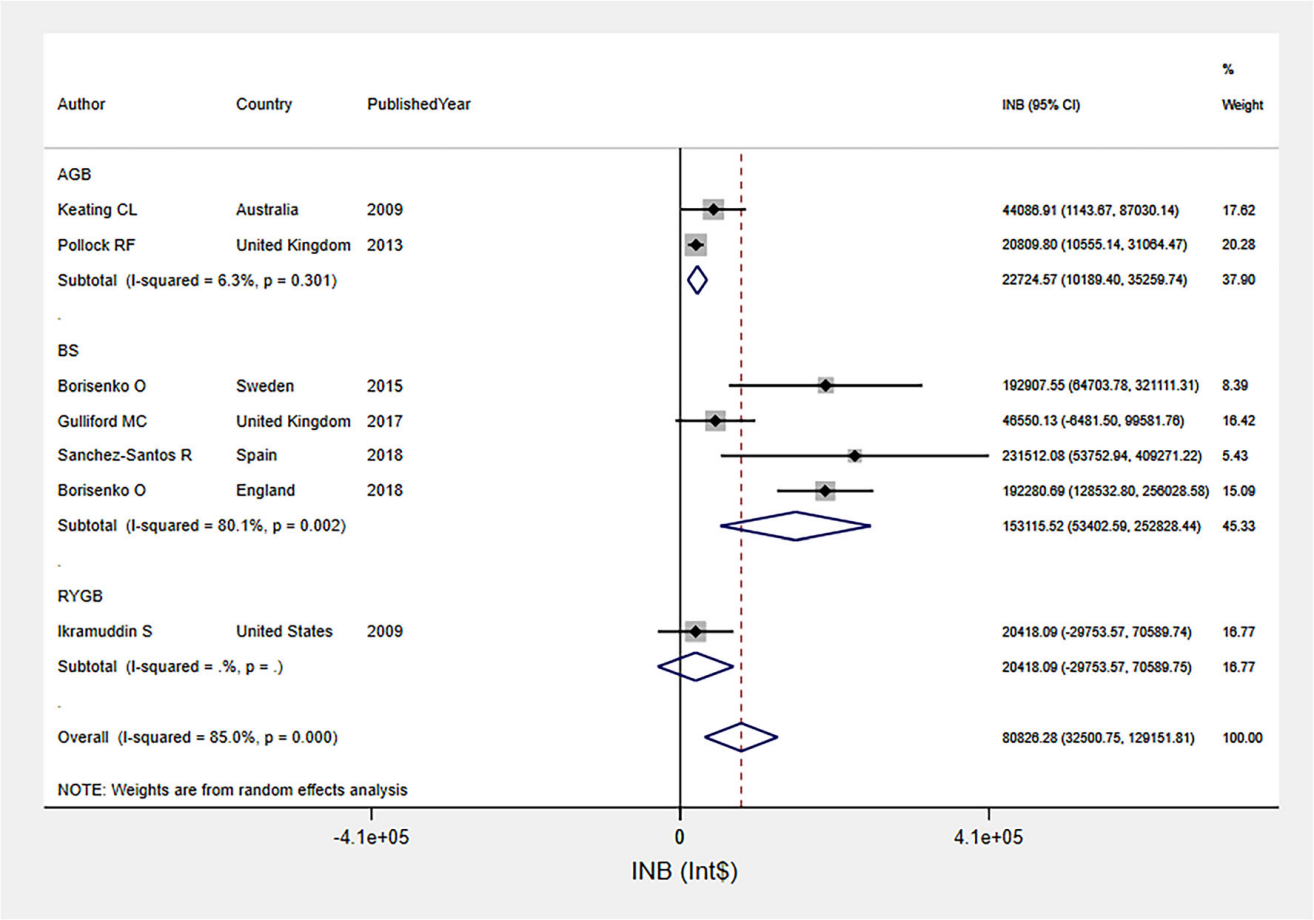
Pooled INBs of bariatric surgery among obesity with diabetes group in high income countries over life-time horizon

The INBs of bariatric surgery among obesity with diabetes group were pooled from seven studies [33, 37, 42, 43, 47, 48, 50] over life-time horizon (see Fig. 4). The pooled INB was $80,826.28 (95% CI $32,500.75, $129,151.81; *I*^2^ = 85.0%), indicating that bariatric surgery was cost-effective relative to usual care. Asymmetry was observed in funnel plot and Egger’s test (coefficient = 2.51, SE = 0.92, *p* = 0.041) (Fig. S4). A contour-enhanced funnel plot (Fig. S5) showed that asymmetry might be caused by heterogeneity.

### INBs of Bariatric Surgery in UMICs

Of the four studies from UMICs [36, 44, 51, 52], three studies [36, 44, 51] with five comparisons were conducted in mixed obesity group, while four comparisons [36, 44, 51, 52] were conducted in obesity with diabetes group [36, 44, 51, 52] (see Table S6). Time horizon varied across studies, i.e., 5 years [51], 10 years [44], 20 years [36], and 50 years [52]. All studies adopted a payer perspective [36, 44, 51, 52]. For mixed group obesity group, the evidence was inconclusive. On the other hand, all studies conducted among obesity with diabetes group revealed that bariatric surgery was cost-effective with the INBs ranging from $4,015.28 to $40,867.97 (see Table S6). Since studies had varying time horizons, the INBs were not pooled.

### Risk of Bias

Risk of bias was assessed using ECOBIAS checklist (see Table S7). All studies had no bias in terms of comparator, outcomes measurement, and discounting. Four studies [27, 31, 45, 46] had unclear risk of wrong model bias. Three studies [45, 46, 51] adopted short-term time-horizon of 5 years, four studies [28, 31, 36, 44] used mid-term period of 10 and 20 years, and 18 studies [25–27, 29, 30, 32–35, 37–43, 48, 49] adopted lifetime horizon. Partial biases related to treatment effect, intermittent data collection, and quality of life weight occurred in almost all studies. Data on long-term treatment effect of bariatric surgery and utility per unit of BMI change were limited. Moreover, half of the studies [25–29, 31, 37, 38, 40, 44–46, 48, 49, 51, 52] were subjected to bias related to internal consistency.

## Discussion

We conducted a systematic review and meta-analysis for pooling INBs of bariatric surgery as compared with usual care. A total of 24 and 4 studies from HICs and UMICs were included in the review, but only data from HICs were sufficiently pooled. We found that bariatric surgery was a cost-effective intervention for mixed obesity group (i.e., with/without diabetes) in HICs over 10-year and lifetime time horizons. In addition, it was a cost-effective intervention for patients with obesity and diabetes in both HICs and UMICs from a payer perspective. For HICs, our analysis provides economic evidences in support of the current clinical practice guideline [53], which recommends bariatric surgery for patients with BMI ≥40 kg/m^2^ and patients with BMI ≥35 kg/m^2^ with co-morbidity.

Comparing the three recent systematic reviews [7, 9, 54], they included 35 unique studies, while our study included 28 studies. Of the 28 included studies, 4 [31, 44, 51, 52] were not included in the previous systematic reviews [7, 9, 54]. Eleven studies [55–65] were included in the previous studies [7, 9, 54] but were not eligible in our present review since they were studies among adolescents (*N* = 4) [61, 63–65], with BMI <32 kg/m^2^ (*N* = 2) [55, 56], with insufficient data for calculating INB (*N* = 3) [57, 59, 62], and studied in a specific group of patients (*N* = 2) [58, 60]. Almost all studies adopted a payer perspective, which considered only direct medical costs, rather than a broader societal perspective. This might be due to the need for evidences among payers to support reimbursement decision while there had been growing demand for bariatric surgery. Notably, a study [29], which was conducted from a societal perspective, indicated that bariatric surgery was cost-effective and that the direct medical costs associated with both the surgery and treatment of comorbidities represented a major cost component.

For mixed obesity group (i.e., with/without diabetes), all evidences from HICs indicated that bariatric surgery was cost-effective over lifetime time horizon. Nevertheless, three studies, which adopted 2-year [27], 5-year [30], and 10-year [31] time horizons, found that bariatric surgery was not cost-effective. Our findings revealed that bariatric surgery might be cost-effective in studies employing a ≥10-year time horizon and more cost-effective over lifetime horizon. This could be possibly explained by the fact that costs for bariatric surgery were driven by high cost of surgery in the first few years [66], while the benefit of significantly lower healthcare expenditures due to the reduction of comorbidities could be observed in the long term after surgery [7]. Most included studies performed bariatric surgery on patients with a mean age ≥40 years. They projected all costs and outcomes incurred after the surgery throughout a lifetime period. It should be noted that INB with a lifetime horizon for these patients can be considered cost-effective as bariatric surgery can help prevent them from comorbidities and complications related to obesity, which finally can improve their health outcomes and decrease healthcare costs. In addition, performing the bariatric surgery with a lifetime horizon among patients with younger ages (i.e., 20 years) was even more cost-effective [38, 40]. This is due to the fact that the young patients continue to gain benefits from surgery for a longer period as compared with their older counterparts.

Nevertheless, there is currently limited evidence on the long-term effects of bariatric surgery. Further studies on the long-term effectiveness and safety of bariatric surgery are warranted to improve the validity of cost-effectiveness studies.

According to our review, bariatric surgery was cost-effective (i.e., positive INB) in all 14 studies (29 comparisons) conducted among obesity with diabetes group. This is similar to a previous systematic review [9], which found that almost all studies (6/7) conducted among obesity with diabetes group indicated that bariatric surgery was cost-effective. Bariatric surgery was generally believed to be more cost-effective in obesity with diabetes group than general obesity group. However, we found that the pooled INB (95% CI) in obesity with diabetes group over lifetime horizon was lower than mixed obesity group but not significant, i.e., $80,826.28 ($32,500.75, $129,151.81) vs. $101,897.96 ($79,390.93, $124,404.99), respectively. It should be noted that remission was higher among patients with recent onset of diabetes and those who were not taking insulin [67, 68]. Less benefit of bariatric surgery was reported in diabetic patients who had already developed complications [67]. Variation in such characteristics of diabetes patients in each included study might possibly lead to the unclear conclusion when comparing the benefits of bariatric surgery between mixed obesity and obesity with diabetes group. Further studies carefully designed to compare cost-effectiveness of bariatric surgery among various characteristics of patients with diabetes (i.e., early diagnosed vs. long-term diabetes) are also warranted.

Presently, many types of bariatric surgery have been used in clinical practice. Nevertheless, most included studies reported mixed types of bariatric surgery. Subgroup analysis by types of bariatric surgery was performed for AGB, SG, and RYGB. We found that SG and AGB were cost-effective when compared with usual care, but not for RYGB. This could be explained by the fact that only three studies for RYGB were included for pooling and they had very large variances. Consequently, the pooled INB has high uncertainty and the interpretation should be made with caution. Based on current evidence, it is still inconclusive on which specific types of bariatric surgery should be selected. Further studies comparing cost-effectiveness among different types of the surgery are thus warranted.

Although this study was the first to provide quantitative evidences on value for money of bariatric surgery by estimating and pooling INB of bariatric surgery, certain limitations were needed to be addressed. First, all included economic evaluation studies were performed in HICs and UMICs, but none was conducted in lower-middle-income countries (LMICs) and low-income countries (LICs). Therefore, future economic evaluation studies of bariatric surgery in such settings should be further investigated. Second, in some cases, where dispersion parameters had not been reported (scenario 5), variances of INBs were adopted from other studies, which had comparable characteristics in terms of country, country income level, characteristics of patients, types of intervention, perspective, discount rate, and time. Nevertheless, adopted dispersion might not fully represent the actual dispersion of the INBs. Third, according to the quality assessment, almost all studies had biases related to treatment effects such that evidences on long-term effects of bariatric surgery were scarce. To improve validity of cost-effectiveness evidences, studies on the long-term treatment effect of bariatric surgery are warranted. Finally, due to data availability, we could only pool the value for money of bariatric surgery among obese patients with diabetes but not with other comorbidities, such as hypertension and obstructive sleep apnea.

In conclusion, our findings indicated that bariatric surgery seems to be cost-effective over 10-year and lifetime horizons in HICs for both mixed obesity group (i.e., with/without diabetes) and obesity with diabetes group. The pooled INB for bariatric surgery as compared with usual care in HICs was estimated to be between $81,000 and $102,000 over the lifetime horizon. For UMICs, bariatric surgery seemed to be cost-effective as compared with usual care among obesity with diabetes group with the INBs ranging from $4,000 to $41,000.

## Supplementary Information

ESM 1 (DOCX 8090 kb)

## References

[1] Park CH, Nam SJ, Choi HS (2019). Comparative efficacy of bariatric surgery in the treatment of morbid obesity and diabetes mellitus: a systematic review and network meta-analysis. Obes Surg.

[2] Gloy VL, Briel M, Bhatt DL, Kashyap SR, Schauer PR, Mingrone G, Bucher HC, Nordmann AJ (2013). Bariatric surgery versus non-surgical treatment for obesity: a systematic review and meta-analysis of randomised controlled trials. BMJ.

[3] Colquitt JL, Pickett K, Loveman E, et al. Surgery for weight loss in adults. Cochrane Database Syst Rev. 2014;(8):Cd003641. 10.1002/14651858.CD003641.pub4 PMC9028049 25105982

[4] Cheng J, Gao J, Shuai X, Wang G, Tao K (2016). The comprehensive summary of surgical versus non-surgical treatment for obesity: a systematic review and meta-analysis of randomized controlled trials. Oncotarget..

[5] Chang S-H, Stoll CRT, Song J, Varela JE, Eagon CJ, Colditz GA (2014). Bariatric surgery: an updated systematic review and meta-analysis, 2003–2012. JAMA Surg.

[6] Muller-Stich BP, Senft JD, Warschkow R (2015). Surgical versus medical treatment of type 2 diabetes mellitus in nonseverely obese patients: a systematic review and meta-analysis. Ann Surg.

[7] Xia Q, Campbell JA, Ahmad H, Si L, de Graaff B, Palmer AJ (2020). Bariatric surgery is a cost-saving treatment for obesity—a comprehensive meta-analysis and updated systematic review of health economic evaluations of bariatric surgery. Obes Rev.

[8] Lopes EC, Heineck I, Athaydes G, Meinhardt NG, Souto KE, Stein AT (2015). Is bariatric surgery effective in reducing comorbidities and drug costs? A systematic review and meta-analysis. Obes Surg.

[9] Campbell JA, Venn A, Neil A, Hensher M, Sharman M, Palmer AJ (2016). Diverse approaches to the health economic evaluation of bariatric surgery: a comprehensive systematic review. Obes Rev.

[10] Reed SD (2014). Statistical considerations in economic evaluation: a guide for cardiologists. Eur Heart J.

[11] Hoch JS, Dewa CS (2008). A clinician’s guide to correct cost-effectiveness analysis: think incremental not average. Can J Psychiatry.

[12] McCabe C, Claxton K, Culyer AJ (2008). The NICE cost-effectiveness threshold: what it is and what that means. Pharmacoeconomics..

[13] World Health Organization. Choosing interventions that are cost-effective [Internet]. Geneva: World Health Orgnanization 2014.

[14] Net Monetary Benefit [Internet]. York Health Economics Consortium. 2016. Available from: https://yhec.co.uk/glossary/net-monetary-benefit/.

[15] Crespo C, Monleon A, Díaz W, Ríos M (2014). Comparative efficiency research (COMER): meta-analysis of cost-effectiveness studies. BMC Med Res Methodol.

[16] Haider S, Chaikledkaew U, Thavorncharoensap M, Youngkong S, Islam MA, Thakkinstian A (2019). Systematic review and meta-analysis of cost-effectiveness of rotavirus vaccine in low-income and lower-middle-income countries. Open Forum Infect Dis.

[17] Bagepally BS, Gurav YK, Anothaisintawee T, Youngkong S, Chaikledkaew U, Thakkinstian A (2019). Cost utility of sodium-glucose cotransporter 2 inhibitors in the treatment of metformin monotherapy failed type 2 diabetes patients: a systematic review and meta-analysis. Value Health.

[18] Chaiyakittisopon K, Pattanaprateep O, Ruenroengbun N, et al. Evaluation of the cost-utility of phosphate binders as a treatment option for hyperphosphatemia in chronic kidney disease patients: a systematic review and meta-analysis of the economic evaluations. Eur J Health Econ. 2021; 10.1007/s10198-021-01275-3. 10.1007/s10198-021-01275-3 PMC8166732 33677736

[19] Moher D, Liberati A, Tetzlaff J, Altman DG (2009). Preferred reporting items for systematic reviews and meta-analyses: the PRISMA statement. PLoS Med.

[20] World Economic Outlook Database [Internet]. 2019 [cited 14 November 2019]. Available from: https://www.imf.org/external/pubs/ft/weo/2019/01/weodata/download.aspx.

[21] Adarkwah CC, van Gils PF, Hiligsmann M, Evers SM (2016). Risk of bias in model-based economic evaluations: the ECOBIAS checklist. Expert Rev Pharmacoecon Outcomes Res.

[22] World Bank Country and Lending Groups [Internet]. 2019 [cited 14 November 2019]. Available from: https://datahelpdesk.worldbank.org/knowledgebase/articles/906519-worldbank-country-and-lending-groups.

[23] DerSimonian R, Laird N (1986). Meta-analysis in clinical trials. Control Clin Trials.

[24] Palmer TM, Peters JL, Sutton AJ, Moreno SG (2008). Contour-enhanced funnel plots for meta-analysis. Stata J.

[25] Craig BM, Tseng DS (2002). Cost-effectiveness of gastric bypass for severe obesity. Am J Med.

[26] Campbell J, McGarry LA, Shikora SA, Hale BC, Lee JT, Weinstein MC (2010). Cost-effectiveness of laparoscopic gastric banding and bypass for morbid obesity. Am J Manag Care.

[27] McEwen LN, Coelho RB, Baumann LM, Bilik D, Nota-Kirby B, Herman WH (2010). The cost, quality of life impact, and cost-utility of bariatric surgery in a managed care population. Obes Surg.

[28] Maklin S, Malmivaara A, Linna M, Victorzon M, Koivukangas V, Sintonen H (2011). Cost-utility of bariatric surgery for morbid obesity in Finland. Br J Surg.

[29] Faria GR, Preto JR, Costa-Maia J (2013). Gastric bypass is a cost-saving procedure: results from a comprehensive Markov model. Obes Surg.

[30] Castilla I, Mar J, Valcarcel-Nazco C, Arrospide A, Ramos-Goni JM (2014). Cost-utility analysis of gastric bypass for severely obese patients in Spain. Obes Surg.

[31] Lewis L, Taylor M, Broom J, Johnston KL (2014). The cost-effectiveness of the LighterLife weight management programme as an intervention for obesity in England. Clin Obes.

[32] Wang BC, Wong ES, Alfonso-Cristancho R (2014). Cost-effectiveness of bariatric surgical procedures for the treatment of severe obesity. Eur J Health Econ.

[33] Borisenko O, Adam D, Funch-Jensen P, Ahmed AR, Zhang R, Colpan Z, Hedenbro J (2015). Bariatric surgery can lead to net cost savings to health care systems: results from a comprehensive European decision analytic model. Obes Surg.

[34] Borisenko O, Mann O, Duprée A. Cost-utility analysis of bariatric surgery compared with conventional medical management in Germany: a decision analytic modeling. BMC Surg. 2017;17(1). 10.1186/s12893-017-0284-0 PMC5543597 28774333

[35] Borisenko O, Lukyanov V, Johnsen SP, Funch-Jensen P. Cost analysis of bariatric surgery in Denmark made with a decision-analytic model. Dan Med J. 2017;64(8). 28869031

[36] Cohen RV, Luque A, Junqueira S, Ribeiro RA, Le Roux CW (2017). What is the impact on the healthcare system if access to bariatric surgery is delayed?. Surg Obes Relat Dis.

[37] Gulliford MC, Charlton J, Prevost T, Booth H, Fildes A, Ashworth M, Littlejohns P, Reddy M, Khan O, Rudisill C (2017). Costs and outcomes of increasing access to bariatric surgery: cohort study and cost-effectiveness analysis using electronic health records. Value Health.

[38] James R, Salton RI, Byrnes JM, Scuffham PA (2017). Cost-utility analysis for bariatric surgery compared with usual care for the treatment of obesity in Australia. Surg Obes Relat Dis.

[39] Lucchese M, Borisenko O, Mantovani LG, Cortesi PA, Cesana G, Adam D, Burdukova E, Lukyanov V, di Lorenzo N (2017). Cost-utility analysis of bariatric surgery in Italy: results of decision-analytic modelling. Obes Facts.

[40] Alsumali A, Eguale T, Bairdain S, Samnaliev M (2018). Cost-effectiveness analysis of bariatric surgery for morbid obesity. Obes Surg.

[41] Borisenko O, Lukyanov V, Debergh I, Dillemans B (2018). Cost-effectiveness analysis of bariatric surgery for morbid obesity in Belgium. J Med Econ.

[42] Borisenko O, Lukyanov V, Ahmed AR (2018). Cost-utility analysis of bariatric surgery. Br J Surg.

[43] Sanchez-Santos R, Padin EM, Adam D, Borisenko O, Fernandez SE, Dacosta EC, Fernández SG, Vazquez JT, de Adana JCR, de la Cruz Vigo F (2018). Bariatric surgery versus conservative management for morbidly obese patients in Spain: a cost-effectiveness analysis. Expert Rev Pharmacoecon Outcomes Res.

[44] Assumpção RP, Bahia LR, da Rosa MQM, et al. Cost-utility of gastric bypass surgery compared to clinical treatment for severely obese with and without diabetes in the perspective of the Brazilian Public Health System. Obes Surg. 2019. 10.1007/s11695-019-03957-7 31214966

[45] Ackroyd R, Mouiel J, Chevallier JM, Daoud F (2006). Cost-effectiveness and budget impact of obesity surgery in patients with type-2 diabetes in three European countries. Obes Surg.

[46] Anselmino M, Bammer T, Fernandez Cebrian JM, Daoud F, Romagnoli G, Torres A (2009). Cost-effectiveness and budget impact of obesity surgery in patients with type 2 diabetes in three European countries(II). Obes Surg.

[47] Ikramuddin S, Klingman CD, Swan T, Minshall ME (2009). Cost-effectiveness of Roux-en-Y gastric bypass in type 2 diabetes patients. Am J Manag Care.

[48] Keating CL, Dixon JB, Moodie ML, Peeters A, Bulfone L, Maglianno DJ, O'Brien PE (2009). Cost-effectiveness of surgically induced weight loss for the management of type 2 diabetes: modeled lifetime analysis. Diabetes Care.

[49] Hoerger TJ, Zhang P, Segel JE, Kahn HS, Barker LE, Couper S (2010). Cost-effectiveness of bariatric surgery for severely obese adults with diabetes. Diabetes Care.

[50] Pollock RF, Muduma G, Valentine WJ (2013). Evaluating the cost-effectiveness of laparoscopic adjustable gastric banding versus standard medical management in obese patients with type 2 diabetes in the UK. Diabetes Obes Metab.

[51] Gil-Rojas Y, Garzón A, Lasalvia P, Hernández F, Castañeda-Cardona C, Rosselli D (2019). Cost-effectiveness of bariatric surgery compared with nonsurgical treatment in people with obesity and comorbidity in Colombia. Value Health Reg Issues.

[52] Viratanapanu I, Romyen C, Chaivanijchaya K, Sornphiphatphong S, Kattipatanapong W, Techagumpuch A, Kitisin K, Pungpapong SU, Tharavej C, Navicharern P, Boonchayaanant P, Udomsawaengsup S (2019). Cost-effectiveness evaluation of bariatric surgery for morbidly obese with diabetes patients in Thailand. J Obes.

[53] Di Lorenzo N, Antoniou SA, Batterham RL (2020). Clinical practice guidelines of the European Association for Endoscopic Surgery (EAES) on bariatric surgery: update 2020 endorsed by IFSO-EC, EASO and ESPCOP. Surg Endosc.

[54] Alsumali A, Al-Hawag A, Samnaliev M, Eguale T (2018). Systematic assessment of decision analytic models for the cost-effectiveness of bariatric surgery for morbid obesity. Surg Obes Relat Dis.

[55] Tang Q, Sun Z, Zhang N, Xu G, Song P, Xu L, Tang W (2016). Cost-effectiveness of bariatric surgery for type 2 diabetes mellitus: a randomized controlled trial in China. Medicine..

[56] Song HJ, Kwon JW, Kim YJ, Oh SH, Heo Y, Han SM (2013). Bariatric surgery for the treatment of severely obese patients in South Korea—is it cost effective?. Obes Surg.

[57] Salem L, Devlin A, Sullivan SD, Flum DR (2008). Cost-effectiveness analysis of laparoscopic gastric bypass, adjustable gastric banding, and nonoperative weight loss interventions. Surg Obes Relat Dis.

[58] Neff R, Havrilesky LJ, Chino J, O'Malley DM, Cohn DE (2015). Bariatric surgery as a means to decrease mortality in women with type I endometrial cancer – an intriguing option in a population at risk for dying of complications of metabolic syndrome. Gynecol Oncol.

[59] Lee YY, Veerman JL, Barendregt JJ (2013). The cost-effectiveness of laparoscopic adjustable gastric banding in the morbidly obese adult population of Australia. PLoS One.

[60] Klebanoff MJ, Corey KE, Chhatwal J, Kaplan LM, Chung RT, Hur C (2017). Bariatric surgery for nonalcoholic steatohepatitis: a clinical and cost-effectiveness analysis. Hepatology..

[61] Klebanoff MJ, Chhatwal J, Nudel JD, Corey KE, Kaplan LM, Hur C (2017). Cost-effectiveness of bariatric surgery in adolescents with obesity. JAMA Surg.

[62] Kim DD, Arterburn DE, Sullivan SD, Basu A (2018). Economic value of greater access to bariatric procedures for patients with severe obesity and diabetes. Med Care.

[63] Chang SH, Stoll CR, Colditz GA (2011). Cost-effectiveness of bariatric surgery: should it be universally available?. Maturitas.

[64] Bairdain S, Samnaliev M (2015). Cost-effectiveness of adolescent bariatric surgery. Cureus.

[65] Ananthapavan J, Moodie M, Haby M, Carter R (2010). Assessing cost-effectiveness in obesity: laparoscopic adjustable gastric banding for severely obese adolescents. Surg Obes Relat Dis.

[66] Smith VA, Arterburn DE, Berkowitz TSZ, et al. Association between bariatric surgery and long-term health care expenditures among veterans with severe obesity. JAMA Surg. 2019:e193732. 10.1001/jamasurg.2019.3732 PMC6822094 31664427

[67] Busetto L (2015). Timing of bariatric surgery in people with obesity and diabetes. Ann Transl Med.

[68] Jans A, Naslund I, Ottosson J, Szabo E, Naslund E, Stenberg E (2019). Duration of type 2 diabetes and remission rates after bariatric surgery in Sweden 2007–2015: a registry-based cohort study. PLoS Med.

