# Supplementary material for: Incremental Net Monetary Benefit of Bariatric Surgery: Systematic Review and Meta-Analysis of Cost-Effectiveness Evidences
Source: Obes Surg. 2021 Apr 24;31(7):3279–90. doi: 10.1007/s11695-021-05415-9 (PMC8175295; doi:10.1007/s11695-021-05415-9)
Supplement: Supplementary file 1 — (DOCX 8090 kb) [file 11695_2021_5415_MOESM1_ESM.docx]

**Supporting information for the manuscript**

**Incremental Net Monetary Benefit of Bariatric Surgery: Systematic Review and Meta-Analysis of Cost-effectiveness Evidences**

Prapaporn Noparatayaporn, Montarat Thavorncharoensap, Usa Chaikledkaew, Bhavani Shankara Bagepally, Ammarin Thakkinstian

**Table S1** PRISMA checklist

| **Section/topic** | **#** | **Checklist item** | **Reported on page #** |
| --- | --- | --- | --- |
| **TITLE** |  |  |  |
| Title | 1 | Identify the report as a systematic review, meta-analysis, or both. | 1 |
| **ABSTRACT** |  |  |  |
| Structured summary | 2 | Provide a structured summary including, as applicable: background; objectives; data sources; study eligibility criteria, participants, and interventions; study appraisal and synthesis methods; results; limitations; conclusions and implications of key findings; systematic review registration number. | 1 |
| **INTRODUCTION** |  |  |  |
| Rationale | 3 | Describe the rationale for the review in the context of what is already known. | 2 |
| Objectives | 4 | Provide an explicit statement of questions being addressed with reference to participants, interventions, comparisons, outcomes, and study design (PICOS). | 2 |
| **METHODS** |  |  |  |
| Protocol and registration | 5 | Indicate if a review protocol exists, if and where it can be accessed (e.g., Web address), and, if available, provide registration information including registration number. | 4 |
| Eligibility criteria | 6 | Specify study characteristics (e.g., PICOS, length of follow-up) and report characteristics (e.g., years considered, language, publication status) used as criteria for eligibility, giving rationale. | 4 |
| Information sources | 7 | Describe all information sources (e.g., databases with dates of coverage, contact with study authors to identify additional studies) in the search and date last searched. | 4 |
| Search | 8 | Present full electronic search strategy for at least one database, including any limits used, such that it could be repeated. | 3, table S2 |
| Study selection | 9 | State the process for selecting studies (i.e., screening, eligibility, included in systematic review, and, if applicable, included in the meta-analysis). | 4 |
| Data collection process | 10 | Describe method of data extraction from reports (e.g., piloted forms, independently, in duplicate) and any processes for obtaining and confirming data from investigators. | 4-5 |
| Data items | 11 | List and define all variables for which data were sought (e.g., PICOS, funding sources) and any assumptions and simplifications made. | 4-5 |
| Risk of bias in individual studies | 12 | Describe methods used for assessing risk of bias of individual studies (including specification of whether this was done at the study or outcome level), and how this information is to be used in any data synthesis. | 5 |
| Summary measures | 13 | State the principal summary measures (e.g., risk ratio, difference in means). | 5 |
| Synthesis of results | 14 | Describe the methods of handling data and combining results of studies, if done, including measures of consistency (e.g., I^2^) for each meta-analysis. | 5 |
| Risk of bias across studies | 15 | Specify any assessment of risk of bias that may affect the cumulative evidence (e.g., publication bias, selective reporting within studies). | 5-6 |
| Additional analyses | 16 | Describe methods of additional analyses (e.g., sensitivity or subgroup analyses, meta-regression), if done, indicating which were pre-specified. | 5-6 |
| **RESULTS** |  |  |  |
| Study selection | 17 | Give numbers of studies screened, assessed for eligibility, and included in the review, with reasons for exclusions at each stage, ideally with a flow diagram. | 6, Figure1 |
| Study characteristics | 18 | For each study, present characteristics for which data were extracted (e.g., study size, PICOS, follow-up period) and provide the citations. | 6-7, Table1, Table S3 |
| Risk of bias within studies | 19 | Present data on risk of bias of each study and, if available, any outcome level assessment (see item 12). | 8-9, Table S7 |
| Results of individual studies | 20 | For all outcomes considered (benefits or harms), present, for each study: (a) simple summary data for each intervention group (b) effect estimates and confidence intervals, ideally with a forest plot. | Figure 2-4, Table S4-6 |
| Synthesis of results | 21 | Present results of each meta-analysis done, including confidence intervals and measures of consistency. | 7-8 |
| Risk of bias across studies | 22 | Present results of any assessment of risk of bias across studies (see Item 15). | 7-8 |
| Additional analysis | 23 | Give results of additional analyses, if done (e.g., sensitivity or subgroup analyses, meta-regression [see Item 16]). | 7-8 |
| **DISCUSSION** |  |  |  |
| Summary of evidence | 24 | Summarize the main findings including the strength of evidence for each main outcome; consider their relevance to key groups (e.g., healthcare providers, users, and policy makers). | 9-12 |
| Limitations | 25 | Discuss limitations at study and outcome level (e.g., risk of bias), and at review-level (e.g., incomplete retrieval of identified research, reporting bias). | 12 |
| Conclusions | 26 | Provide a general interpretation of the results in the context of other evidence, and implications for future research. | 12 |
| **FUNDING** |  |  |  |
| Funding | 27 | Describe sources of funding for the systematic review and other support (e.g., supply of data); role of funders for the systematic review. | - |

**Table S2** Search terms used in five databases

| **Database** | **Word search** | **Items found** |
| --- | --- | --- |
| Pubmed | ((((((((((obese) OR obesity)) OR obes*)) OR ((((Overweight) OR Overweights)) OR Overweight*)))) AND (((((((((((((("mini-gastric bypass") OR (("long-limb gastric bypass") OR "very long-limb gastric bypass")) OR "Roux-en-Y gastric bypass")) OR "Gastric Bypass")) OR "gastric banding") OR "sleeve gastrectomy") OR "Vertical Banded Gastroplasty") OR "intragastric balloon") OR "biliopancreatic diversion") OR "duodenal switch")) OR (((((("Bariatric Surgery") OR "Bariatric Surgery"[Mesh])) OR "metabolic surgery")) OR "Weight loss surgery"))) AND (((((("incremental cost effectiveness ratio") OR ICER)) OR ((cost) OR "incremental cost")) OR (((((((("disability adjusted life year") OR "disability adjusted life years")) OR ((DALY) OR DALYs))) OR (((("incremental disability adjusted life year") OR "incremental disability adjusted life years")) OR (("incremental DALY") OR "incremental DALYs")))) OR ((((((QALY) OR QALYs)) OR (("Quality-Adjusted Life Years") OR "Quality-Adjusted Life Year"))) OR (((("incremental QALY") OR "incremental QALYs")) OR (("incremental Quality-Adjusted Life Years") OR "incremental Quality-Adjusted Life Year"))))) OR (("incremental net benefit") OR INB))) AND (((((("Economic Evaluation") OR "Economic Evaluations")) OR "Cost-benefit") OR "Cost-effectiveness") OR "Cost-utility") | 290 |
| Scopus | ( ( ALL ( obes* )  OR  ALL ( overweight*  ) ) )  AND  ( ( ALL ( "gastric bypass" )  OR  ALL ( "gastric banding" )  OR  ALL ( "sleeve gastrectomy" )  OR  ALL ( "Vertical Banded Gastroplasty" )  OR  ALL ( "intragastric balloon" )  OR  ALL ( "biliopancreatic diversion" )  OR  ALL ( "duodenal switch" )  OR  ALL ( "Bariatric Surgery" )  OR  ALL ( "metabolic surgery" )  OR  ALL ( "weight loss surgery" ) ) )  AND  ( ( ( ( ( ( ALL ( "Quality-Adjusted Life Year" ) )  OR  ( ALL ( "Quality-Adjusted Life Years" ) ) )  OR  ( ( ALL ( qaly ) )  OR  ( ALL ( qalys ) ) ) )  OR  ( ( ( ALL ( "incremental Quality-Adjusted Life Year"    ) )  OR  ( ALL ( "incremental Quality-Adjusted Life Years"    ) ) )  OR  ( ( ALL ( "incremental QALY" ) )  OR  ( ALL ( "incremental QALYs" ) ) ) ) )  OR  ( ( ( ( ALL ( "disability adjusted life year" ) )  OR  ( ALL ( "disability adjusted life years" ) ) )  OR  ( ( ALL ( daly ) )  OR  ( ALL ( dalys ) ) ) )  OR  ( ( ( ALL ( "incremental DALY" ) )  OR  ( ALL ( "incremental DALYs" ) ) )  OR  ( ( ALL ( "incremental disability adjusted life year" ) )  OR  ( ALL ( "incremental disability adjusted life years" ) ) ) ) ) )  OR  ( ( ALL ( "incremental net benefit" ) )  OR  ( ALL ( inb ) ) )  OR  ( ( ALL ( "incremental cost effectiveness ratio" ) )  OR  ( ALL ( icer ) ) )  OR  ( ( ALL ( cost  ) )  OR  ( ALL ( "incremental cost"    ) ) ) )  AND  ( ( ALL ( "Economic Evaluation" ) )  OR  ( ALL ( "Cost Benefit" ) )  OR  ( ALL ( "Cost-effectiveness" ) )  OR  ( ALL ( "Cost-utility" ) ) ) | 3,562 |
| Cochrane | MeSH descriptor: [Obesity, Morbid] explode all trees or MeSH descriptor: [Obesity] explode all trees or obes* and MeSH descriptor: [Bariatric Surgery] explode all trees or bariatric surgery or Weight loss surgery or metabolic surgery or Gastric bypass or Gastrectomy or Gastroplasty or Biliopancreatic Diversion or gastric banding or duodenal switch or Gastric balloon or intragastric balloon and incremental net benefit or INB or MeSH descriptor: [Hospital Costs] explode all trees or MeSH descriptor: [Health Care Costs] explode all trees or MeSH descriptor: [Direct Service Costs] explode all trees or cost or incremental cost effectiveness ratio or ICER or Quality-Adjusted Life Year or MeSH descriptor: [Quality-Adjusted Life Years] explode all trees or QALY or disability adjusted life year or DALY and economic evaluation or "cost benefit" or "cost effectiveness" or "cost utility" | 72 |
| CEA registry | overweight, obese, obesity, weight loss, bariatric, metabolic surgery, Roux-en-Y gastric bypass, gastric bypass, mini-gastric bypass, long-limb gastric bypass, very long-limb gastric bypass, sleeve gastrectomy, gastric banding, Vertical Banded Gastroplasty, intragastric balloon, biliopancreatic diversion, duodenal switch | 135 |
| CRD | (MeSH DESCRIPTOR Obesity EXPLODE ALL TREES) OR (Obesity) OR (Obese) IN DARE, NHSEED, HTA and (((MeSH DESCRIPTOR Bariatric Surgery EXPLODE ALL TREES) OR (Bariatric Surgery)) OR (Metabolic surgery) OR (weight loss surgery)) OR ((duodenal switch) OR (((Gastric Bypass) OR (MeSH DESCRIPTOR Gastric Bypass EXPLODE ALL TREES)) OR (gastric banding) OR (sleeve gastrectomy)) OR ((intragastric balloon) OR ((Vertical Banded Gastroplasty) OR (MeSH DESCRIPTOR Gastroplasty EXPLODE ALL TREES) ) OR ((MeSH DESCRIPTOR Biliopancreatic Diversion EXPLODE ALL TREES) OR (Biliopancreatic Diversion)))) IN DARE, NHSEED, HTA | 336 |

**Table S3** Characteristics of population

| **Author year** | **Definition of obesity** | **Criteria for bariatric surgery** | **Targeted population** | **Base case** | | | |
| --- | --- | --- | --- | --- | --- | --- | --- |
|  |  |  |  | **Age (years)** | **BMI (kg/m^2^)** | **Female (%)** | **Co-morbidity (%)** |
| **Population: Mixed obesity group** | | | | | | | |
| Craig BM 2002 [1] | - | BMI >40 kg m^2^ without CVD, drug addictions, major psychological disorders and non-smoking who failed from non-surgical weight loss. | BMI 40-50 kg/m^2^ without chronic medical conditions | 35-55 | 40-50 | - | - |
| Campbell J 2010 [2] | Obese: BMI 30-34.9 kg/m^2^, morbidly obese I: BMI 35-39.9 kg/m^2^), morbidly obese II: BMI 40-49.9 kg/m^2^, and super obese: BMI >50 kg/m^2^ | BMI ≥40/≥35 kg/m^2^  with comorbid | BMI ≥40/≥ 35 kg/m^2^ with comorbid | 18-74 | - | - | - |
| McEwen LN 2010 [3] | - | BMI ≥40/≥35 kg/m^2^ with comorbid | BMI ≥40/≥ 35 kg/m^2^ with comorbid | 44 | 52 | 90.0 | HT (49), hyperlipidemia (41), DM (36), obstructive sleep apnea (49), gastro esophageal reflux disease (42), arthritis (46), depression (60) |
| Maklin S 2011 [4] | - | BMI ≥40/≥35 kg/m^2^ with significant obesity-related comorbidity | Morbid obesity (Base case: mean BMI 47 kg/m^2^) | 43 | 47 | - | DM, sleep apnea |
| Faria GR 2013 [5] | - | BMI >40/>35 kg/m^2^  with comorbid | Baseline: mean BMI 49.6 kg/m^2^ | 40 | 49.6 | - | - |
| Castilla I 2014 [6] | - | - | BMI ≥40/≥ 35 kg/m^2^ with comorbid | 30 | 50.7 | 87.3 | DM (20.25) |
| Lewis L 2014 [7] | - | BMI ≥40/>35 kg m^2^ with comorbidities (e.g. DM, HT) who failed from non-surgical weight loss for at least 6 months and could be improved with weight loss. | BMI ≥40 kg/m^2^ | Starting age 45.54 | 44.56 | - | - |
| Wang BCM 2014 [8] | - | BMI ≥40/≥35 kg/m^2^ with comorbid | BMI ≥ 40/≥ 35 kg/m^2^ with comorbid | 53 | 44 | 100 | - |
| Borisenko O 2015 [9] | - | - | BMI ≥40/≥ 35 kg/m^2^ with DM | 41 | 42.8 | 76 | DM (18.39), HT |
| Borisenko O 2017a [10] | - | BMI >40 kg/m2 who failed from conservative management or BMI>35 kg/m^2^ with DM | BMI ≥40/≥35 kg/m^2^ with DM | 40.4 | 48.8 | 73.7 | DM (20.6), HT |
| Borisenko O 2017b [11] | - | - | Base case: mean BMI 42 kg/m^2^ | 40 | 42 | 77.4 | DM (23.2) |
| Cohen RV 2017 [12] | - | - | BMI >35 kg/m^2^ with/without DM | 41 | 48.6 | 79 | DM (22.3), HT |
| Gulliford MC 2017 [13] | - | BMI >40 kg/m^2^ or who have been diagnosed with comorbidities e.g. DM, HT, CVD, osteoarthritis, dyslipidemia or sleep apnea | Severe (BMI 35–39 kg/m^2^)/morbid obesity with/without DM | 46 | - | 50 | DM (19), coronary heart disease (4) |
| James R 2017 [14] | Class I: 30–34.9 kg/m^2^, class II: 35–39.9 kg/m^2^, class III: ≥40 kg/m^2^ | - | Base case: BMI >35 kg/m^2^ | 30 | >35 | 100 | - |
| Lucchese M 2017 [15] | - | BMI ≥40/35-40 kg/m^2^ with comorbid (e.g. metabolic, cardiopulmonary, mental diseases, joint problems) which could be improved after surgery | BMI ≥40/≥ 35 kg/m^2^ with DM | 40.6 | 46.2 | 75.36 | DM (20), HT |
| Alsumali A 2018 [16] | - | Class III (BMI ≥40 kg/m^2^)/class II (BMI 35.0–39.9 kg/m^2^) obesity with obesity-related comorbidities that failed from nonsurgical interventions | BMI ≥35 kg/m^2^ | 18-74 | - | 78 | - |
| Borisenko O 2018a [17] | - | - | BMI ≥40/≥35 kg/m^2^ with DM | 39.2 | 41.44 | 79.31 | DM (9.17), HT |
| Borisenko O 2018b [18] | - | BMI >35 kg/m^2^ with DM or another obesity-related co-morbidity, BMI >40 kg/m^2^ who fail from non-surgical methods for at 6 months, BMI >50 kg/m^2^, BMI ≥30 kg/m^2^ who have recent onset of DM | BMI ≥40/≥35 kg/m^2^ with DM | 45.4 | 50.5 | 76 | DM (30), HT |
| Sanchez-Santos R 2018 [19] | - | - | BMI ≥40/≥35 kg/m^2^ with DM | 41.1 | 47.56 | 80.5 | DM (18.6), HT |
| Assumpção RP 2019 [20] | - | BMI >40/>35 kg/m^2^ with ≥1 obesity-related comorbidities | BMI > 40/> 35 kg/m^2^ with ≥1 obesity-related comorbidities | - | - | - | DM |
| **Population: Obesity with diabetes group** | | | | | | | |
| Ackroyd R 2006 [21] | - | BMI ≥35 kg/m^2^  with DM who failed from ≥1 year with well conducted medical treatment | BMI ≥35 kg/m^2^ with DM | - | - | - | DM (100) |
| Anselmino M 2009 [22] | - | BMI ≥35 kg/m^2^  with DM who failed from ≥1 year with well conducted medical treatment and in the absence of contraindication | BMI ≥35 kg/m^2^ with DM | - | - | - | DM (100) |
| Ikramuddin S 2009 [23] | BMI ≥30 kg/m^2^ | BMI ≥40/35 kg/m^2^ with major comorbidity such as DM after failure of a 1-year course of well-conducted medical treatment | Severely obese with DM (Base case: mean BMI 48.4 kg/m^2^) | 50.1 | 48.4 | 77.9 | HT, hyperlipidemia, CVD, renal disease, retinopathy, peripheral neuropathy, foot ulcer, amputation, cataract, macular edema, severe vision loss, and DM (average 8.7 years of DM) |
| Keating CL 2009 [24] | - | - | Base case: BMI 37 kg/m^2^ with DM (< 2 years) | 49 | 37 | - | DM (100) |
| Hoerger TJ 2010 [25] | - | - | BMI ≥35 kg/m^2^ with DM | 35-74 | ≥35 | - | DM (100) (<5, ≥10 years of DM) |
| Pollock RF 2013 [26] | - | BMI >40 kg/m^2^ or who have been diagnosed with comorbidities e.g. DM, HT, CVD, osteoarthritis, dyslipidemia, sleep apnea | Base case: BMI 37.1 kg/m^2^ with DM (average 1 year) | 46.9 | 37.1 | 53.5 | DM (100) (1 year of DM), kidney disease, CVD, ocular complication, neuropathy |
| Gil-Rojas Y 2019 [27] | BMI ≥30 kg/m^2^ | BMI ≥40 kg/m^2^ with/without comorbidities or 35-40 kg/m^2^ with comorbidities that could improve with weight loss | BMI >35 kg/m^2^ with ≥1 comorbidity | 40 | 45.6 | - | DM (100), HT, dyslipidemia, sleep apnea |
| Viratanapanu I 2019 [28] |  | Morbidly obese with DM who have inadequate diabetes control with only medical treatment | BMI >32.5 kg/m^2^ with DM | 41.8 | 50.1 | 58.9 | DM (100), HT |

BMI: Body Mass Index; DM: Diabetes Mellitus; CVD: cardiovascular disease; HT: hypertension

**Table S4** Incremental net monetary benefit (INB) of bariatric surgery among mixed obesity group in high income countries

| **Author year** | **Intervention** | **Scenario** | **CE Threshold (Int$)** | **ICER (Int$) per QALY** | **INB (Int$)*** |
| --- | --- | --- | --- | --- | --- |
| **Time horizon: Lifetime** | | | | | |
| Craig BM 2002 [1] | RYGB | 5 | 50,000.00 | 23,660.93 | 53,318.12 |
| Campbell J 2010 [2] | AGB | 4 | 25,000.00 | 6,898.71 | 36,999.05 |
|  | RYGB | 4 | 25,000.00 | 7,154.21 | 51,734.94 |
| McEwen LN 2010 [3] | BS | 5 | 50,000.00 | 1,769.69 | 84,885.35 |
| Faria GR 2013 [5] | RYGB | 5 | 32,894.74 | -12,137.00 | 85,560.31 |
|  | AGB | 5 | 32,894.74 | -4,113.20 | 22,574.84 |
| Castilla I 2014 [6] | RYGB | 4 | 46,224.96 | -4,060.59 | 283,107.65 |
| Wang BCM 2014 [8] | RYGB | 5 | 50,000.00 | 7,793.09 | 118,179.34 |
|  | AGB | 5 | 50,000.00 | 7,320.79 | 93,894.27 |
| Borisenko O 2015 [9] | BS | 4 | 34,731.95 | -2,160.20 | 151,257.82 |
| Borisenko O 2017a [10] | BS | 4 | 45,278.14 | -3,725.61 | 156,812.00 |
| Borisenko O 2017b [11] | BS | 1 | 30,066.06 | -1,801.87 | 92,417.00 |
| Gulliford MC 2017 [13] | BS | 1 | 28,943.56 | 11,360.87 | 43,967.78 |
| James R 2017 [14] | AGB | 4 | 48,746.52 | 18,403.29 | 15,171.61 |
|  | RYGB | 4 | 48,746.52 | 17,041.90 | 22,510.28 |
|  | SG | 4 | 48,746.52 | 20,712.92 | 21,585.9 |
| Lucchese M 2017 [15] | BS | 4 | 68,775.79 | -3,856.07 | 232,421.96 |
| Alsumali A 2018 [16] | SG | 5 | 100,000.00 | 8,212.91 | 127,584.06 |
|  | RYGB | 5 | 100,000.00 | 5,842.91 | 178,898.47 |
|  | AGB | 5 | 100,000.00 | 8,812.65 | 84,804.24 |
| Borisenko O 2018a [17] | BS | 4 | 36,630.04 | -2,509.38 | 195,697.08 |
| Borisenko O 2018b [18] | BS | 4 | 43,415.34 | -1,076.26 | 177,966.42 |
| Sanchez-Santos R 2018 [19] | BS | 4 | 46,224.96 | -108.58 | 203,867.60 |
| **Time horizon: 10 years** | | | | | |
| Maklin S 2011 [4] | BS | 5 | 43,987.02** | -50,822.89 | 54,989.75 |
| Castilla I 2014 [6] | RYGB | 3 | 46,224.96 | 6,604.72 | 17,908.35 |
| Lewis L 2014 [7] | AGB | 5 | 28,943.56 | 33,503.20 | -1,924.17 |
|  | RYGB | 5 | 28,943.56 | 26,685.53 | 1,652.88 |
| Lucchese M 2017 [15] | BS | 5 | 68,775.79 | 3,441.17 | 71,868.08 |
| Borisenko O 2017a [10] | BS | 5 | 45,278.14 | 3,437.25 | 50,209.06 |
| Borisenko O 2017b [11] | BS | 1 | 30,066.06 | 2,557.66 | 30,259.24 |
| Borisenko O 2018a [17] | BS | 1 | 36,630.04 | 3,776.71 | 42,709.33 |
| Borisenko O 2018b [18] | BS | 1 | 43,415.34 | 5,171.72 | 65,014.15 |
| Sanchez-Santos R 2018 [19] | BS | 5 | 46,224.96 | 9,501.30 | 58,757.85 |
| **Time horizon: <10 years** | | | | | |
| Castilla I 2014 [6] (5 years) | RYGB | 3 | 46,224.96 | -43,082.87 | -11,342.09 |
| McEwen LN 2010 [3] (2 years) | BS | 5 | 50,000.00 | 60,432.75 | -2,921.17 |

BS: Mixed types of bariatric surgery; AGB: adjustable gastric banding; RYGB: Roux-en-Y gastric bypass; SG: sleeve gastrectomy

All the monetary units’ values were adjusted to international dollar (Int$) for the year 2019

* Compared with non-bariatric surgery, ** 1 GDP per capita was adopted

**Table S5** Incremental net monetary benefit (INB) of bariatric surgery among obesity with diabetes group in high income countries

| **Author year** | **Intervention** | **Scenario** | **CE Threshold (Int$)** | **ICER (Int$) per QALY** | **INB (Int$)*** |
| --- | --- | --- | --- | --- | --- |
| **Time horizon: lifetime** | | | | | |
| Ikramuddin S 2009 [23] | RYGB | 3 | 50,000.00 | 27,288.00 | 20,418.09 |
| Keating CL 2009 [24] | AGB | 5 | 34,818.94 | -1,920.15 | 44,086.91 |
| Hoerger TJ 2010 [25] | AGB(newly DM) | 4 | 50,000.00 | 15,291.61 | 54,492.17 |
|  | RYGB(newly DM) | 4 | 50,000.00 | 9,075.27 | 90,443.65 |
|  | AGB(established DM) | 4 | 50,000.00 | 13,883.24 | 48,396.46 |
|  | RYGB (established DM) | 4 | 50,000.00 | 12,573.78 | 63,624.58 |
| Pollock RF 2013 [26] | AGB | 2 | 28,943.56 | 6,324.21 | 20,809.80 |
| Borisenko O 2015 [9] | BS | 5 | 34,731.95 | -4,239.27 | 192,907.54 |
| Gulliford MC 2017 [13] | BS | 5 | 28,943.56 | 9,842.15 | 46,550.13 |
| Borisenko O 2018 [18] | BS | 5 | 43,415.34 | -4,061.37 | 192,280.69 |
| Sanchez-Santos R 2018 2018 [19] | BS | 5 | 46,224.96 | -2,514.42 | 231,512.08 |
| **Time horizon: 10 years** | | | | | |
| Borisenko O 2018 [18] | BS | 5 | 43,415.34 | -34.84 | 175,973.21 |
| Sanchez-Santos R 2018 [19] | BS | 5 | 46,224.96 | 4,626.93 | 66,556.85 |
| **Time horizon: 5 years** | | | | | |
| Ackroyd R 2006 [21] | RYGB (German) | 5 | 45,120.20** | -5,960.63 | 68,448.30 |
|  | AGB (German) | 5 | 45,120.20** | -5,538.27 | 52,178.22 |
|  | RYGB (France) | 5 | 44,682.86** | -6,710.41 | 68,866.99 |
|  | AGB (France) | 5 | 44,682.86** | -6,667.56 | 52,890.94 |
|  | RYGB (UK) | 5 | 28,943.56 | 3,049.15 | 34,698.50 |
|  | AGB (UK) | 5 | 28,943.56 | 3,877.27 | 25,818.28 |
| Anselmino M 2009 [22] | RYGB (Austria) | 5 | 35,928.14 | -2,107.32 | 50,941.76 |
|  | AGB (Austria) | 5 | 35,928.14 | -4,166.58 | 41,229.87 |
|  | RYGB (Italy) | 5 | 41,265.47 | -1,951.70 | 57,923.50 |
|  | AGB (Italy) | 5 | 41,265.47 | -1,686.98 | 44,148.91 |
|  | RYGB (Spain) | 5 | 46,224.96 | 2,558.50 | 58,516.99 |
|  | AGB (Spain) | 5 | 46,224.96 | 4,681.21 | 42,713.60 |

BS: Mixed types of bariatric surgery; AGB: adjustable gastric banding; RYGB: Roux-en-Y gastric bypass; SG: sleeve gastrectomy

All the monetary units’ values were adjusted to international dollar (Int$) for the year 2019

* Compared with non-bariatric surgery, ** 1 GDP per capita was adopted

**Table S6** Incremental net monetary benefit (INB) of bariatric surgery among obesity groups in upper middle income countries

| **Author year** | **Intervention** | **Time horizon (years)** | **Scenario** | **CE Threshold (Int$)** | **ICER (Int$) per QALY** | **INB (Int$)*** |
| --- | --- | --- | --- | --- | --- | --- |
| **Population: Mixed obesity group** | | | | | | |
| Cohen RV 2017 [12] | BS (with/without DM) | 20 | 5 | 16,574.73** | 739.18 | 27,712.22 |
| Assumpção RP 2019 [20] | ORYGB  (obese without DM) | 10 | 4 | 16,705.10** | 2,079.91 | 18,427.74 |
| Gil-Rojas Y 2019 [27] | BS  (obese with hypertension) | 5 | 4 | 14,259.84*** | 37,498.93 | -3,114.04 |
|  | BS  (obese with dyslipidemia) | 5 | 4 | 14,259.84*** | -134,245.67 | -5,821.42 |
|  | BS  (obese with sleep apnea) | 5 | 4 | 14,259.84*** | -4,866.30 | 6,770.65 |
| **Population: Obesity with diabetes group** | | | | | | |
| Cohen RV 2017 [12] | BS | 20 | 5 | 16,574.73** | -653.86 | 32,217.48 |
| Assumpção RP 2019 [20] | ORYGB | 10 | 4 | 16,705.10** | 1,953.46 | 27,733.09 |
| Gil-Rojas Y 2019 [27] | BS | 5 | 4 | 14,259.84*** | 5,317.11 | 4,015.28 |
| Viratanapanu I 2019 [28] | BS | 50 | 5 | 16,685.88*** | 2,244.90 | 40,867.97 |

BS: Mixed types of bariatric surgery; AGB: adjustable gastric banding; RYGB: Roux-en-Y gastric bypass; SG: sleeve gastrectomy

All the monetary units’ values were adjusted to international dollar (Int$) for the year 2019

* Compared with non-bariatric surgery, ** 1 GDP per capita was adopted, *** Studies applied GDP-based threshold

**Table S7** ECOBIAS checklist

|  | Craig BM 2002 [1] | Ackroyd R 2006 [21] | Anselmino M 2009 [22] | Ikramuddin S 2009 [23] | Keating CL 2009 [24] | Campbell J 2010 [2] | Hoerger TJ 2010 [25] | McEwen LN 2010 [3] | Maklin S 2011 [4] | Faria GR 2013 [5] | Pollock RF 2013 [26] | Castilla I 2014 [6] | Lewis L 2014 [7] | Wang BCM 2014 [8] |
| --- | --- | --- | --- | --- | --- | --- | --- | --- | --- | --- | --- | --- | --- | --- |
| **PART A. Overall checklist for bias in economic evaluation** | | | | | | | | | | |  |  |  |  |
| 1. Narrow perspective bias | P | P | P | P | P | P | U | U | P | Y | P | P | P | P |
| 2.Inefficient comparator bias* | Y | Y | Y | Y | Y | Y | Y | Y | Y | Y | Y | Y | Y | Y |
| 3.Cost measurement omission bias | P | P | P | P | P | P | P | P | P | U | P | P | P | P |
| 4.Intermittent data collection bias | P | P | P | P | Y | P | P | P | P | U | Y | P | U | U |
| 5.Invalid valuation bias | P | Y | Y | U | Y | Y | P | P | P | U | Y | P | Y | U |
| 6.Ordinal ICER bias | Y | Y | Y | Y | Y | Y | Y | Y | Y | Y | Y | Y | Y | Y |
| 7.Double-counting bias | Y | Y | Y | Y | Y | Y | Y | Y | Y | U | Y | Y | Y | Y |
| 8.Inappropriate discounting bias | Y | Y | Y | Y | Y | Y | Y | Y | Y | Y | Y | Y | Y | Y |
| 9.Limited sensitivity analysis bias§ | P | P | P | P | P | Y | P | P | P | P | Y | P | P | P |
| 10.Sponsor bias | N | Y | Y | Y | Y | Y | Y | Y | Y | Y | Y | Y | Y | N |
| 11.Reporting and dissemination bias | NA | NA | NA | NA | NA | NA | NA | NA | NA | NA | NA | NA | NA | NA |
| **PART B. Model-specific aspects of bias in economic evaluation** | | | | | | | | | | |  |  |  |  |
| I Bias related to structure | | | | | | | | | | |  |  |  |  |
| 12.Structural assumptions bias | P | U | U | Y | P | P | P | U | U | Y | Y | Y | P | P |
| 13.No treatment comparator bias* | Y | Y | Y | Y | Y | Y | Y | Y | Y | Y | Y | Y | Y | Y |
| 14.Wrong model bias | P | U | U | Y | Y | Y | Y | U | Y | Y | Y | Y | U | Y |
| 15.Limited time horizon bias | Y | N | N | P | Y | Y | Y | Y | N | Y | P | Y | N | Y |
| II Bias related to data | | | | | | | | | | |  |  |  |  |
| 16.Bias related to data identification | P | P | P | P | P | P | Y | U | P | U | Y | P | P | Y |
| 17.Bias related to baseline data | P | P | P | Y | P | P | Y | U | P | U | Y | P | P | Y |
| 18.Bias related to treatment effects | P | P | P | P | P | P | P | U | P | U | P | P | P | P |
| 19.Bias related to quality-of-life weights (utilities) | P | P | U | P | P | P | U | P | P | U | Y | P | P | Y |
| 20.Non-transparent data incorporation bias | P | P | P | P | P | Y | U | U | P | U | Y | Y | P | P |
| 21.Limited scope bias§ | P | P | P | P | P | Y | P | P | P | P | Y | P | P | P |
| III Bias related to consistency | | | | | | | | | | |  |  |  |  |
| 22.Bias related to internal consistency | N | N | N | Y | N | N | N | N | N | N | Y | Y | N | Y |

Y: Yes; P: Partly; U: Unclear; N: No; NA: Not applicable

**Table S7** ECOBIAS checklist continued

|  | Borisenko O 2015 [9] | Borisenko O 2017a [10] | Borisenko O 2017b [11] | Cohen RV 2017 [12] | Gulliford MC 2017 [13] | James R 2017 [14] | Lucchese M 2017 [15] | Alsumali A 2018 [16] | Borisenko O 2018a [17] | Borisenko O 2018b [18] | Sanchez-Santos R 2018 [19] | Assumpção RP 2019 [20] | Gil-Rojas Y 2019 [27] | Viratanapanu I 2019 [28] |
| --- | --- | --- | --- | --- | --- | --- | --- | --- | --- | --- | --- | --- | --- | --- |
| **PART A. Overall checklist for bias in economic evaluation** | | | | | | | | | |  |  |  |  |  |
| 1.Narrow perspective bias | P | P | P | P | P | P | P | P | P | P | P | P | P | P |
| 2.Inefficient comparator bias* | Y | Y | Y | Y | Y | Y | Y | Y | Y | Y | Y | Y | Y | Y |
| 3.Cost measurement omission bias | P | P | P | P | P | P | P | P | P | P | P | P | P | P |
| 4.Intermittent data collection bias | P | P | P | U | Y | U | P | P | P | P | U | U | U | U |
| 5.Invalid valuation bias | Y | Y | Y | P | Y | U | Y | P | Y | Y | U | Y | P | Y |
| 6.Ordinal ICER bias | Y | Y | Y | Y | Y | Y | Y | Y | Y | Y | Y | Y | Y | Y |
| 7.Double-counting bias | Y | Y | Y | Y | Y | Y | Y | Y | Y | Y | Y | N | Y | N |
| 8.Inappropriate discounting bias | Y | Y | Y | Y | Y | Y | Y | Y | Y | Y | Y | Y | Y | Y |
| 9.Limited sensitivity analysis bias§ | P | P | P | P | P | P | P | P | P | P | P | P | P | P |
| 10.Sponsor bias | Y | Y | Y | Y | Y | U | Y | U | Y | Y | Y | Y | Y | U |
| 11.Reporting and dissemination bias | NA | NA | NA | NA | NA | NA | NA | NA | NA | NA | NA | NA | NA | NA |
| **PART B. Model-specific aspects of bias in economic evaluation** | | | | | | | | | |  |  |  |  |  |
| I Bias related to structure | | | | | | | | | |  |  |  |  |  |
| 12.Structural assumptions bias | Y | Y | Y | P | Y | P | Y | P | Y | Y | Y | P | P | P |
| 13.No treatment comparator bias* | Y | Y | Y | Y | Y | Y | Y | Y | Y | Y | Y | Y | Y | Y |
| 14.Wrong model bias | Y | Y | Y | Y | Y | Y | Y | Y | Y | Y | Y | Y | Y | Y |
| 15.Limited time horizon bias | Y | Y | Y | N | Y | Y | Y | Y | Y | Y | Y | N | N | P |
| II Bias related to data | | | | | | | | | |  |  |  |  |  |
| 16.Bias related to data identification | Y | Y | Y | P | P | P | Y | Y | Y | Y | Y | P | P | P |
| 17.Bias related to baseline data | Y | Y | Y | P | Y | P | Y | P | Y | Y | Y | P | P | Y |
| 18.Bias related to treatment effects | P | P | P | Y | P | P | P | P | P | P | P | P | P | P |
| 19.Bias related to quality-of-life weights (utilities) | P | P | P | P | P | P | P | P | P | P | P | P | P | P |
| 20.Non-transparent data incorporation bias | Y | Y | Y | Y | Y | P | Y | Y | Y | Y | U | Y | P | Y |
| 21.Limited scope bias§ | Y | P | P | P | P | P | P | P | P | P | P | P | P | P |
| III Bias related to consistency | | | | | | | | | |  |  |  |  |  |
| 22.Bias related to internal consistency | Y | Y | Y | Y | N | N | Y | N | Y | Y | Y | N | N | N |

Y: Yes; P: Partly; U: Unclear; N: No; NA: Not applicable

**
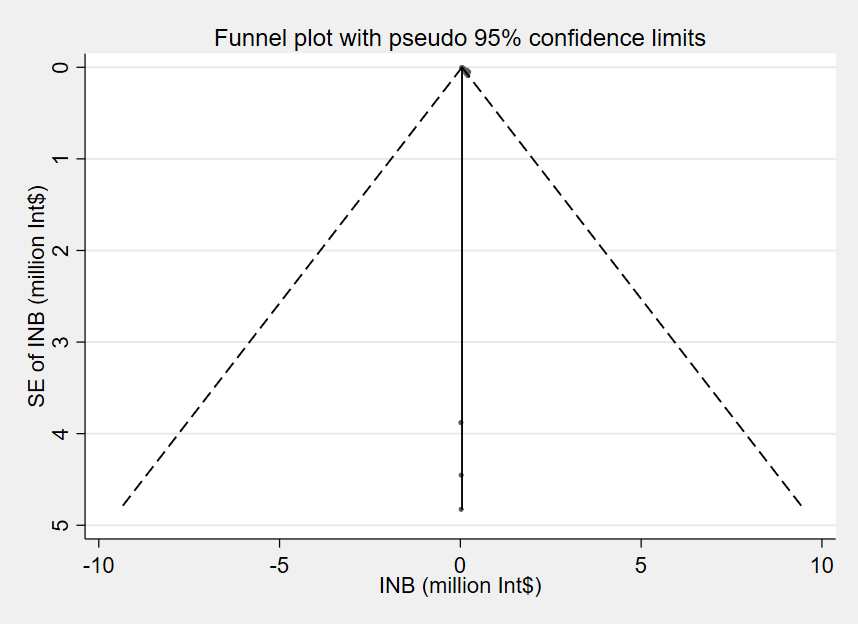
**

**Figure S1** Funnel plot among mixed obesity group in high income countries under payer perspective and lifetime horizon

**
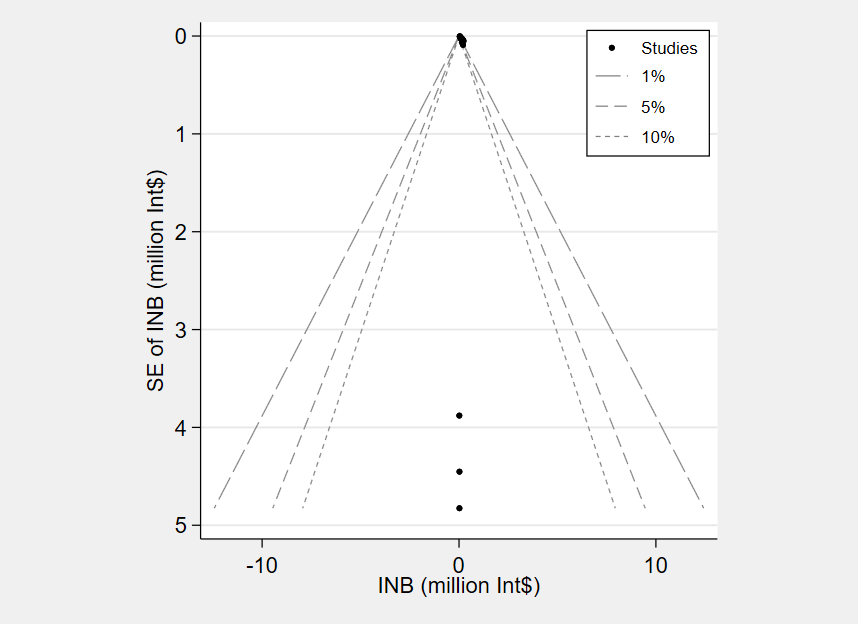
**

**Figure S2** Contour enhanced funnel plot among mixed obesity group in high income countries under payer perspective and lifetime horizon

**
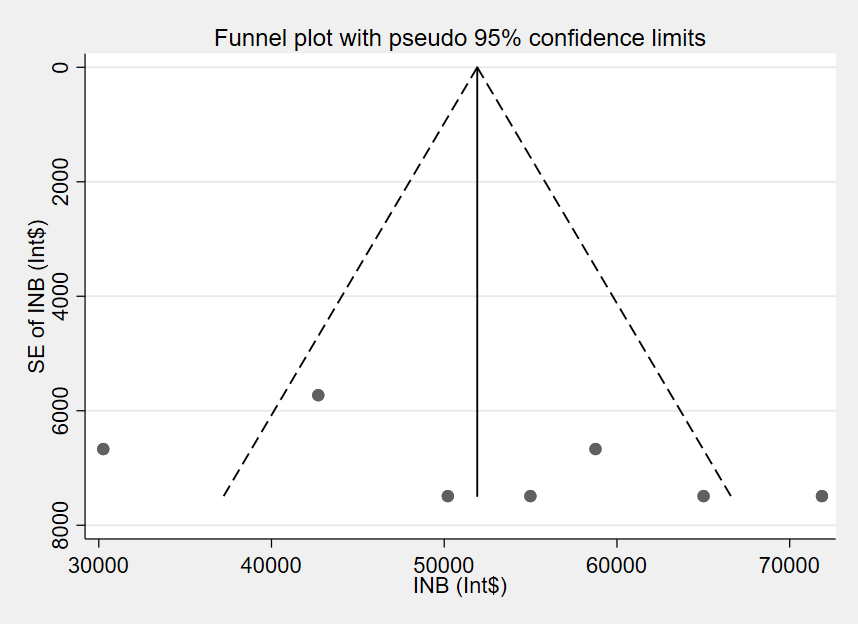
**

**Figure S3** Funnel plot among mixed obesity group in high income countries under payer perspective and 10-year time horizon


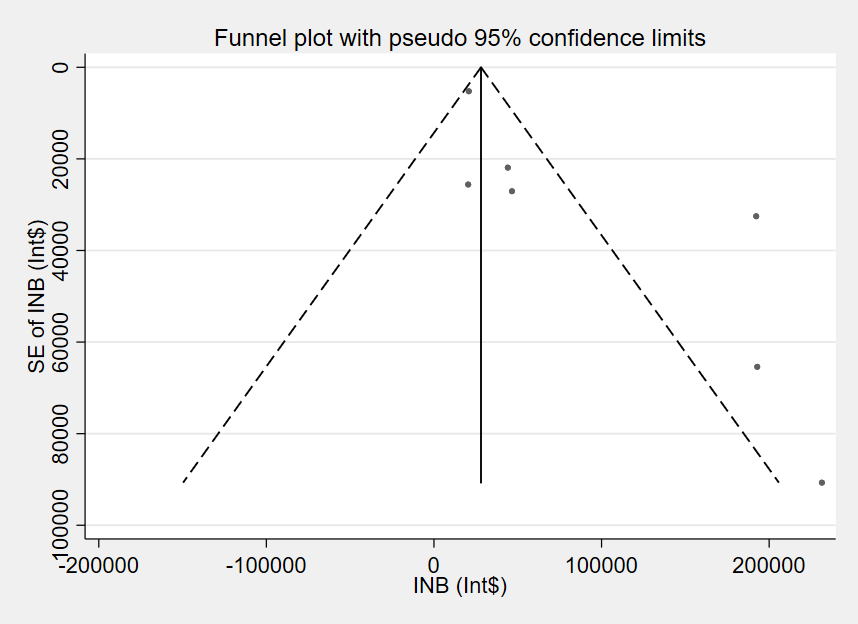


**Figure S4** Funnel plot among obesity with diabetes group in high income countries under payer perspective and lifetime horizon


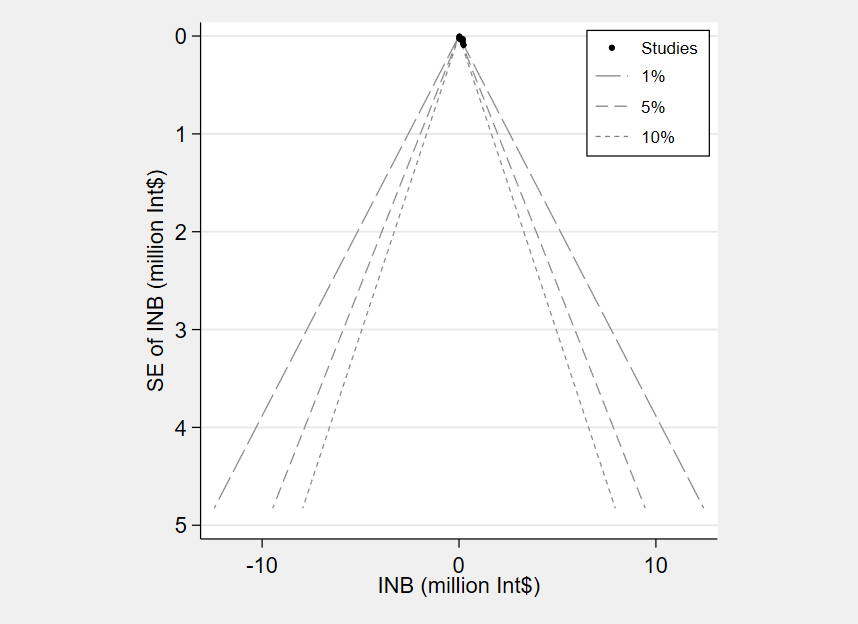


**Figure S5** Contour enhanced funnel plot among obesity with diabetes group in high income countries under payer perspective and lifetime horizon

**Reference**

1. Craig BM, Tseng DS. Cost-effectiveness of gastric bypass for severe obesity. Am J Med. 2002;113(6):491-8.

2. Campbell J, McGarry LA, Shikora SA, Hale BC, Lee JT, Weinstein MC. Cost-effectiveness of laparoscopic gastric banding and bypass for morbid obesity. Am J Manag Care. 2010;16(7):e174-87.

3. McEwen LN, Coelho RB, Baumann LM, Bilik D, Nota-Kirby B, Herman WH. The cost, quality of life impact, and cost-utility of bariatric surgery in a managed care population. Obes Surg. 2010;20(7):919-28.

4. Maklin S, Malmivaara A, Linna M, Victorzon M, Koivukangas V, Sintonen H. Cost-utility of bariatric surgery for morbid obesity in Finland. Br J Surg. 2011;98(10):1422-9.

5. Faria GR, Preto JR, Costa-Maia J. Gastric bypass is a cost-saving procedure: results from a comprehensive Markov model. Obes Surg. 2013;23(4):460-6.

6. Castilla I, Mar J, Valcarcel-Nazco C, Arrospide A, Ramos-Goni JM. Cost-utility analysis of gastric bypass for severely obese patients in Spain. Obes Surg. 2014;24(12):2061-8.

7. Lewis L, Taylor M, Broom J, Johnston KL. The cost-effectiveness of the LighterLife weight management programme as an intervention for obesity in England. Clin Obes. 2014;4(3):180-8.

8. Wang BCM, Wong ES, Alfonso-Cristancho R, et al. Cost-effectiveness of bariatric surgical procedures for the treatment of severe obesity. European Journal of Health Economics. 2014;15(3):253-63.

9. Borisenko O, Adam D, Funch-Jensen P, et al. Bariatric Surgery can Lead to Net Cost Savings to Health Care Systems: Results from a Comprehensive European Decision Analytic Model. Obesity Surgery. 2015;25(9):1559-68.

10. Borisenko O, Mann O, Duprée A. Cost-utility analysis of bariatric surgery compared with conventional medical management in Germany: A decision analytic modeling. BMC Surgery. 2017;17(1).

11. Borisenko O, Lukyanov V, Johnsen SP, Funch-Jensen P. Cost analysis of bariatric surgery in Denmark made with a decision-analytic model. Danish Medical Journal. 2017;64(8).

12. Cohen RV, Luque A, Junqueira S, Ribeiro RA, Le Roux CW. What is the impact on the healthcare system if access to bariatric surgery is delayed? Surgery for Obesity and Related Diseases. 2017;13(9):1619-27.

13. Gulliford MC, Charlton J, Prevost T, et al. Costs and Outcomes of Increasing Access to Bariatric Surgery: Cohort Study and Cost-Effectiveness Analysis Using Electronic Health Records. Value in Health. 2017;20(1):85-92.

14. James R, Salton RI, Byrnes JM, Scuffham PA. Cost-utility analysis for bariatric surgery compared with usual care for the treatment of obesity in Australia. Surgery for Obesity and Related Diseases. 2017;13(12):2012-20.

15. Lucchese M, Borisenko O, Mantovani LG, et al. Cost-Utility Analysis of Bariatric Surgery in Italy: Results of Decision-Analytic Modelling. Obes Facts. 2017;10(3):261-72.

16. Alsumali A, Eguale T, Bairdain S, Samnaliev M. Cost-Effectiveness Analysis of Bariatric Surgery for Morbid Obesity. Obesity Surgery. 2018;28(8):2203-14.

17. Borisenko O, Lukyanov V, Debergh I, Dillemans B. Cost-effectiveness analysis of bariatric surgery for morbid obesity in Belgium. Journal of Medical Economics. 2018;21(4):365-73.

18. Borisenko O, Lukyanov V, Ahmed AR. Cost-utility analysis of bariatric surgery. Br J Surg. 2018.

19. Sanchez-Santos R, Padin EM, Adam D, et al. Bariatric surgery versus conservative management for morbidly obese patients in Spain: a cost-effectiveness analysis. Expert Rev Pharmacoecon Outcomes Res. 2018;18(3):305-14.

20. Assumpção RP, Bahia LR, da Rosa MQM, et al. Cost-Utility of Gastric Bypass Surgery Compared to Clinical Treatment for Severely Obese With and Without Diabetes in the Perspective of the Brazilian Public Health System. Obesity Surgery. 2019.

21. Ackroyd R, Mouiel J, Chevallier JM, Daoud F. Cost-effectiveness and budget impact of obesity surgery in patients with type-2 diabetes in three European countries. Obes Surg. 2006;16(11):1488-503.

22. Anselmino M, Bammer T, Fernandez Cebrian JM, Daoud F, Romagnoli G, Torres A. Cost-effectiveness and budget impact of obesity surgery in patients with type 2 diabetes in three European countries(II). Obes Surg. 2009;19(11):1542-9.

23. Ikramuddin S, Klingman CD, Swan T, Minshall ME. Cost-effectiveness of Roux-en-Y gastric bypass in type 2 diabetes patients. American Journal of Managed Care. 2009;15(9):607-15.

24. Keating CL, Bulfone L, Dixon JB, et al. Cost-effectiveness of surgically induced weight loss for the management of type 2 diabetes: Modeled lifetime analysis. Diabetes Care. 2009;32(4):567-74.

25. Hoerger TJ, Zhang P, Segel JE, Kahn HS, Barker LE, Couper S. Cost-effectiveness of bariatric surgery for severely obese adults with diabetes. Diabetes Care. 2010;33(9):1933-9.

26. Pollock RF, Muduma G, Valentine WJ. Evaluating the cost-effectiveness of laparoscopic adjustable gastric banding versus standard medical management in obese patients with type 2 diabetes in the UK. Diabetes Obes Metab. 2013;15(2):121-9.

27. Gil-Rojas Y, Garzón A, Lasalvia P, Hernández F, Castañeda-Cardona C, Rosselli D. Cost-Effectiveness of Bariatric Surgery Compared With Nonsurgical Treatment in People With Obesity and Comorbidity in Colombia. Value in Health Regional Issues. 2019;20:79-85.

28. Viratanapanu I, Romyen C, Chaivanijchaya K, et al. Cost-Effectiveness Evaluation of Bariatric Surgery for Morbidly Obese with Diabetes Patients in Thailand. Journal of Obesity. 2019;2019.
